# Comparison of RNA-seq and microarray platforms for splice event detection using a cross-platform algorithm

**DOI:** 10.1186/s12864-018-5082-2

**Published:** 2018-09-25

**Authors:** Juan P. Romero, María Ortiz-Estévez, Ander Muniategui, Soraya Carrancio, Fernando J. de Miguel, Fernando Carazo, Luis M. Montuenga, Remco Loos, Rubén Pío, Matthew W. B. Trotter, Angel Rubio

**Affiliations:** 1 CEIT and Tecnun, University of Navarra, Parque Tecnológico de San Sebastián, Paseo Mikeletegi 48, 20009 San Sebastián, Gipuzkoa Spain; 2 Celgene Institute for Translational Research Europe, Celgene Corporation, Parque Científico y Tecnológico Cartuja 93, Centro de Empresas Pabellón de Italia, Isaac Newton, 4, E-41092 Seville, Spain; 3 Program in Solid Tumors and Biomarkers, CIMA, University of Navarra, Avda. Pío XII, 55, E-31008 Pamplona, Navarra Spain; 4 Department of Histology and Pathology, University of Navarra, Campus Universitario, 31009 Pamplona, Navarra Spain; 5 IdiSNA, Navarra Institute for Health Research, Recinto de Complejo Hospitalario de Navarra, Irunlarrea 3, 31008 Pamplona, Navarra Spain; 6 Department of Biochemistry and Genetics, University of Navarra, Campus Universitario, 31009 Pamplona, Navarra Spain; 7 CIBERONC, Centro de Investigación Biomédica en Red, Instituto de Salud Carlos III, Calle Monforte de Lemos 3-5, Pabellón 11. Planta 0, 28029 Madrid, Spain

**Keywords:** Alternative splicing, RNA-seq, Microarrays

## Abstract

**Background:**

RNA-seq is a reference technology for determining alternative splicing at genome-wide level. Exon arrays remain widely used for the analysis of gene expression, but show poor validation rate with regard to splicing events. Commercial arrays that include probes within exon junctions have been developed in order to overcome this problem.

We compare the performance of RNA-seq (Illumina HiSeq) and junction arrays (Affymetrix Human Transcriptome array) for the analysis of transcript splicing events. Three different breast cancer cell lines were treated with CX-4945, a drug that severely affects splicing. To enable a direct comparison of the two platforms, we adapted EventPointer, an algorithm that detects and labels alternative splicing events using junction arrays, to work also on RNA-seq data. Common results and discrepancies between the technologies were validated and/or resolved by over 200 PCR experiments.

**Results:**

As might be expected, RNA-seq appears superior in cases where the technologies disagree and is able to discover novel splicing events beyond the limitations of physical probe-sets. We observe a high degree of coherence between the two technologies, however, with correlation of EventPointer results over 0.90. Through decimation, the detection power of the junction arrays is equivalent to RNA-seq with up to 60 million reads.

**Conclusions:**

Our results suggest, therefore, that exon-junction arrays are a viable alternative to RNA-seq for detection of alternative splicing events when focusing on well-described transcriptional regions.

**Electronic supplementary material:**

The online version of this article (10.1186/s12864-018-5082-2) contains supplementary material, which is available to authorized users.

## Background

Alternative Splicing (AS) is known to play a major role in human biology, and the identification of transcriptional splicing patterns has potential uses for diagnosis, prognosis, and therapeutic target evaluation in the disease context [1, 2]. The development of exon microarrays enabled the transcriptomic study of differential splicing events, but PCR validation rates for identification of splice differences via microarray analysis tend to be lower than those observed for identification of differential gene expression using similar technologies [3–5]. Junction arrays [6–10] have been proposed to overcome this problem by using oligonucleotide probe-sets that interrogate junctions between exons in the transcriptome, as well as the exons themselves.

Since the advent of next-generation sequencing (NGS), RNA-seq has become the technology of choice via which to detect and quantify alternative splicing (for a review see [11]). Various published works compare the performance of RNA-seq and expression microarrays for the analysis of gene expression [12, 13], but a thorough evaluation of both technologies in terms of their ability to detect differential AS events has yet to be presented. In the present study, we perform a comparison of RNA-seq technology (using the Illumina HiSeq platform) and junction arrays commercialized by Affymetrix (Human Transcriptome array, or HTA).

AS can be studied from two complementary points of view: with focus on transcripts or splicing events respectively. In the former, the subject of analysis is the transcript (or isoform), whereas in the latter, the subject(s) are the splicing events themselves.

The pipeline of the transcript-focused approach uses RNA-seq data with [14] and without known annotations in order to reconstruct the transcriptome and estimate the concentration values of the transcripts. Finally, the significance of change in absolute or relative concentrations is assessed using suitable statistical methods [15–17]. Transcript reconstruction is challenging [18] (even the better methods display transcriptome reconstruction levels below 50% when using simulated reads) and any error in reconstruction of transcript structure may be propagated to the output of statistical analysis. Moreover, the challenge of estimating isoform concentrations for genes with many transcripts yields wide confidence intervals [19].

On this basis, therefore, an event-based method appears a more suitable approach via which to compare AS detection technologies, with the additional benefit of straightforward validation using PCR. Event-based methods focus directly on the analysis of differential splicing events, rather than first attempting to estimate transcript concentration levels. These events can be classified into five canonical categories [20]: cassette exon, alternative 3′, alternative 5′, mutually exclusive exons and intron retention. In some cases, alternative start and termination sites are included also when defining splicing events. This approach has gained traction and several algorithms have been developed recently for detection of splicing events using RNA-seq data, including rMats, SplAdder, spliceGrapher or SGSeq [21–24]. SpliceGrapher and SGSeq detect events prior to application of separate software in order to state corresponding statistical significance, whereas rMats and SplAdder perform both detection and statistical analysis. Alongside NGS-based approaches, AS event detection methods are available for exon arrays [25], and exon-junction arrays [6, 8, 9, 26]. The latter methods display validation rates well above 50%.

The principal aim of this work is to compare RNA-seq and exon-junction microarray technologies in their ability to detect differential AS events. To do so comprehensively, and to allow as close to a direct comparison as possible, we have adapted the EventPointer [8] algorithm for application to data from both platforms, generated from the same control experiment. The control experiment comprises three distinct triple-negative breast cancer (TNBC) cell-lines, exposed in culture to a drug known to affect the transcriptional machinery and, thereby, to induce AS events.

Further to comparative analysis of the resulting data, we conclude that both technologies show considerable concordance with high PCR validation rates, and that exon-junction microarrays have potential as an alternative to RNA-seq profiling for detection of AS events in annotated transcripts.

## Results

CX-4945 is a potent and selective orally bioavailable small molecule inhibitor of casein kinase CK2 [27], which has been proposed previously as a cancer therapy [28], and which has been shown to regulate splicing in mammalian cells [29]. RNA samples taken from three distinct triple-negative breast cancer (TNBC) cell-lines, exposed to CX-4945 and also to a DMSO control, were profiled using both RNA sequencing^1^ and hybridization to exon-junction microarrays (see Methods for details). We extended the EventPointer algorithm (available via Bioconductor, see Methods) for application to data from both platforms and applied it to the corresponding datasets in order to identify AS events.

Prior to the comparison of platforms for splice event detection, the data was assessed at the gene level in order to ensure signal quality and coherence. Gene expression was computed from RNA-seq data using Kallisto [14] to quantify expression as the sum of isoform concentrations for each gene. RMA [30] was used to quantify gene expression from microarray data, using annotation files from Brainarray [31]. The same version of the Ensembl Transcriptome (Ensembl v.74, GRCh 37.75) was used in both cases.

Considering each technology independently, correlation between sample replicate profiles in each cell-line and experimental condition is high for both platforms (correlation coefficient ranging from 0.988 to 0.996 in arrays and 0.996 to 0.997 in RNA-seq). When comparing profiles from the same samples between technologies, strong coherence is observed for well-expressed genes. Median correlation of gene expression between technologies on the same samples is 0.510, and gene expression patterns across all samples display correlation of 0.680 between technologies. The first one is smaller owing to the different probe affinities of the set of probes that interrogates each gene. When only the 50% most highly expressed genes are considered, the median correlation of gene expression patterns is 0.750 (Additional file 1). The gene expression correlations observed are similar to previously reported comparisons between RNA-seq and exon arrays [32]. It is important to point out that the expected correlations for gene expression are larger (either using microarrays or RNAseq) since the number of probes/reads that interrogate a gene is larger than the ones that interrogate a splicing event.

### Events detected by RNA-seq and junction arrays show strong qualitative and quantitative concordance, with a subset detected exclusively by one of the technologies

**Fig. 1:**
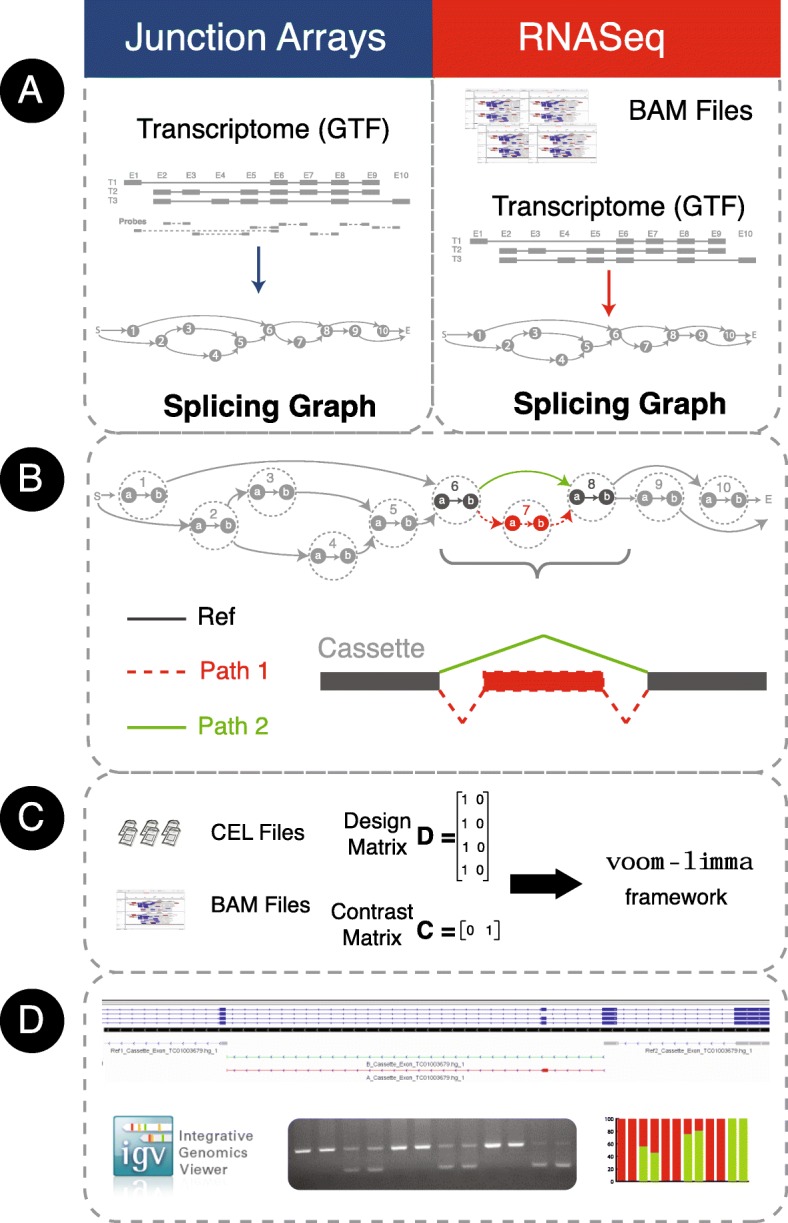
EventPointer overview for junction arrays and RNA-Seq data. **a** The CEL or BAM files are the input data for each technology. The splicing graph for each gene is built using the array annotation files or directly using the sequenced reads. **b** Each node in the splicing graph is splitted into two nodes that correspond to the start and end positions in the genome respectively. EventPointer identifies events within each gene and annotates the type of event. In the figure, among the events in the gene, an exon cassette is highlighted. **c** Statistical significance of the events is computed. **d** Finally, the top-ranked events are validated using PCR and the results visualized in IGV

Figure 1 depicts the EventPointer pipeline for both profiling technologies (see original publication [8] for further detail), with CEL files (microarray) or BAM files (RNA-seq) as starting input. When building the splicing graph, each exon is split into two nodes that correspond to its start and end genomic positions respectively (Fig. 1b). Each event is described by two alternative paths (Paths 1 and 2) and a shared reference path (Path Ref) within the splicing graph. These paths are sets of edges in the splicing graph. Paths 1 and 2 are mutually exclusive in terms of isoforms (i.e. if an isoform includes Path 1 it does not include Path 2 and vice versa) and all isoforms interrogated by the event share the reference path. Therefore, events are contained in several isoforms (at least two). A simple example would be the cassette exon shown in Fig. 1b: the reference path is composed by the edge that links nodes 6a and 6b (i.e. the coverage of exon 6 or the signal in the probe-set of the array that interrogates this exon) and the edge that links nodes 8a and 8b (coverage of exon 8). All these measurements are summarized into one average value. Path 1 includes the edges in the path 6b-7a-7b-8a (coverage of exon 7 and its flanking junctions) and Path 2 is the edge that links 6b and 8a (coverage of the skipping junction). EventPointer distinguishes between events as corresponding to: cassettes; alternative 5′; alternative 3′; mutually exclusive exons; alternative first exons; and alternative end exons. Complex events that do not match any of these categories are denoted as such.

Three different cell-lines were profiled, each exposed to CX-4945 and DMSO respectively across five replicates. AS differences were tested using a linear model which controlled for cell-line differences. Using the read coverage (or probe-set signal) for each path, a statistical analysis based on voom-limma [17, 33] is applied to determine the significance of each event via comparison between alternative path signals (see Methods for details). In addition to the statistical analysis, we compute the Percent Splice Index (PSI or Ψ) [34], an estimate of relative isoform concentrations that map to paths 1 and 2 for each event. In a cassette event, if the exon is retained, Ψ is equal to one. If it is skipped, Ψ is equal zero. If both isoforms that retain and skip the exon are present, Ψ is the ratio between the expression of the isoforms that retain the exon and the overall expression of the isoforms that skip or retain the exon. Ψ has become the standard method to quantify splicing events.

In order to identify well-expressed events (more likely to be biologically significant and less prone to validation error), the comparison of AS detection was performed on a subset of the data with expression above a set threshold (see Methods for details). In brief, a junction coverage threshold was applied to the RNA-seq data (default 2 FPKM) and a threshold on expression percentile applied to the microarray data (default probe-set expression greater than 25% of probes in any sample profile).

Table 1 displays the number of detected events after setting a threshold on the expression for both technologies, the number of differentially spliced events (*p* value < 0.001) with their corresponding False Discovery Rate (FDR) detected via application of EventPointer to RNA-seq and microarray data respectively. The statistical analysis compares differential AS in profiles from cell-lines treated with CX-4945 and with DMSO control. As may be expected, setting more stringent expression thresholds yields fewer events detected with better FDR on both platforms. The FDR is the estimated proportion of false discoveries (i.e. events are assumed to have differential splicing and that do not). For example, an FDR of 5e-4 means that 0.05% of the selected events are expected to be false positives.

**Table 1:** Number and statistical significance of detected AS events using both RNA-seq and array technologies

Expression Threshold	Detected Events	Significant events	FDR for significant
RNASeq			
Junction coverage > 6 FPKM	9277	4526	2.7e-4
Junction coverage > 2 FPKM	34,961	13,780	4.7e-4
Junction coverage > 2/3 FPKM	92,986	29,443	7.0e-4
Exon-junction arrays			
Signal > 50%	10,114	2385	9.2e-4
Signal > 25%	31,506	6197	1.37e-3
No threshold	92,405	11,761	3.45e-3

for different expression thresholds, default filters are junction coverage greater than 2 FPKM for RNA-seq and probe-set signal greater than top 25% quantile for microarray

Table 1 shows that fixing *p*-value to 1e-3 yields False Discovery Rates (FDRs) less than 1% for both technologies. The expected proportion of AS events appears high (1-π_0_ approx. 46%) [35], i.e. more than 46% of the events have its splicing patterns altered, which reflects the anticipated strong effect of compound exposure on the splicing machinery. It is also apparent that, for a similar number of detected AS events, the FDR corresponding to RNA-seq analysis is smaller.

Events detected by both technologies (referred to as “matched events” hereon) were defined by a stringent criterion in which nucleotide sequences of paths identified via one technology must be a subset of sequences identified via the other, yielding 6222 matched events. When reporting correspondence and divergence between AS events, below, the following naming convention is used: R^+^ represents number of events deemed significantly altered in RNA-seq analysis (*p* value <1e-3); R^−^ represents number of events deemed not significantly altered in RNA-seq analysis (*p* value > 0.2). M^+^ and M^−^ are the counterpart terms used to describe microarray results. Events not detected by each technology are labelled R∅ and M∅ respectively.

A subset of matched events is significant in both technologies (R^+^M^+^) and shows coherent change in the corresponding Ψ. There are also significant events detected by only one of the technologies (R^+^M∅ and R∅M^+^). The summary of findings is presented in Table 2.

**Table 2:** Number of AS events detected per technology, alongside statistical significance of events against distinct thresholds

Matched Events					
Matched Events	Significant in both (R^+^M^+^)		FDR (RNASeq)	FDR (arrays)	
6222	1324		4.96e-4	6.23e-4	
R^+^M∅			R∅M^+^		
Expression Threshold	Detected Events	FDR	Expression Threshold	Detected Events	FDR for significant
Junction coverage > 6 FPKM	2973	2.44e-4	Signal > 50%	1016	1.46e-3
Junction coverage > 2 FPKM	10,617	4.56e-4	Signal > 25%	3297	1.99e-3
Junction coverage > 2/3 FPKM	25,063	6.90e-4	No threshold	7581	4.85e-3

Where thresholds not shown, default filters were employed (junction coverage > 2 FPKM for RNA-seq; upper quartile probe signal for microarrays)

Table 2 shows that the FDR of the events detected only by RNA-seq is similar to that for events detected by both platforms (4.56e-4 vs. 4.96e-4). In other words, the reliability of events discovered only by RNA-seq is similar to that of events identified by both technologies. In the case of the arrays, the FDR of matched events is three times smaller than for those discovered solely by the arrays (1.99e-3 vs 6.23e-4 i.e. R∅M^+^ events are less reliable than R^+^M^+^ events for the same *p*-value threshold. In addition, Table 2 shows that the number of significant events that are RNA-seq specific (R^+^M∅) is larger than the number of significant events detected only by arrays (R∅M^+^) (10,617 vs 3297 events).

**Fig. 2:**
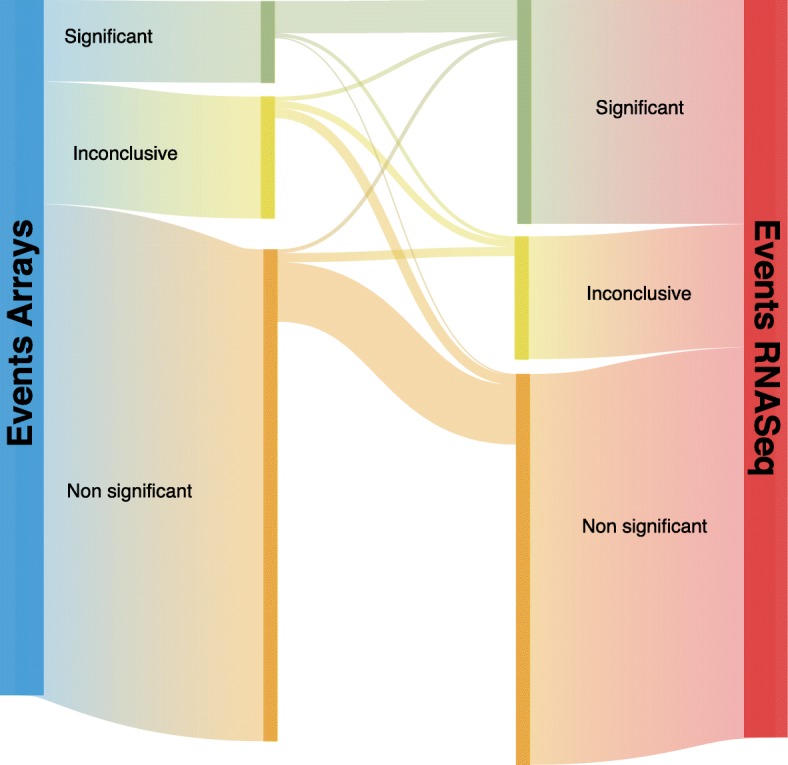
Correspondence between the events detected by arrays and RNA-seq. An event is considered to be significant if the p.value is smaller than 0.001 and non-significant is it is larger than 0.2. Events with *p*-values between both are considered to be inconclusive cases

Figure 2 depicts a Sankey diagram of the relationship between matched events. An event is declared to be significant (in either technology) if the *p* value is smaller than 1e-3. It is declared non-significant is the *p* value is larger than 0.2 and inconclusive otherwise. It is apparent that many events that are significant for RNA-seq are not detected by arrays, but also that events significantly detected via arrays are not detected by RNAseq. Most matched events are consistent across technologies: significant events for one technology are also significant for the other.

**Fig. 3:**
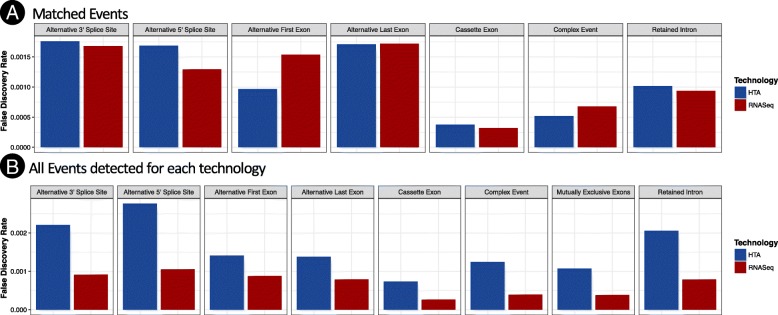
FDR for different types of events using both technologies. Panel **a** shows the FDR for matched events. Panel **b** shows FDR for the events detected in each technology regardless or being matched or not. In both technologies, alternative 3′, 5′, first and last exons have larger FDR than other types of events

We also considered the FDR for different types of splicing events in both technologies. As shown in Fig. 3, alternative 3′ (5′), start and end sites have larger FDR than cassette exons., i.e. they are harder to measure. There were too few matched mutually exclusive events to estimate accurately FDR for this type of events.

### PCR validation rates are over 80% in both technologies

PCR validation was performed on a subset of predicted AS events drawn from each of the subsets discussed in previous sections, i.e. events detected by one or both technologies. PCRs were performed on:

1. Five top-ranked events detected by both technologies (*topRNA* and *topArrays*) regardless of the matching with the other technology
2. Five top-ranked events detected by one technology (R^+^M∅ and R∅M^+^)
3. Five top-ranked events significant in one technology (R^+^M^−^ and R^−^M^+^)
4. Five top ranked events detected by both technologies (R^+^M^+^)

These potential 35 validations are in fact 29 since there is overlap in the top-ranked events of different categories. The characteristics of validated events (genome location, event type, etc.) and links to the corresponding PCR images are included in Additional file 2. PCR for events in non-coherent classes (R^+^M^−^, R^−^M^+^) required up to 40 PCR cycles and were harder to validate in general. The corresponding GTF files to browse these events in IGV [36] are included in Additional file 3. All the results are summarized in Table 3.

**Table 3:** PCR validation for RNA-seq and microarray technologies across events detected by one or both technologies

AS Event Category	RNA-seq	Arrays
Top-ranked events (topRNA, topArrays)	5/5	5/5
Significant in RNA-seq and not detected by arrays (R + M∅)	5/5	–
Significant in arrays and not detected by RNA-seq (R∅M+)	–	5/5
Detected by both. Significant in RNA-seq, not significant in arrays (R + M-)	5/5	
Detected by both. Significant in arrays, not significant in RNA-seq (R-M+)	3/5	
Detected by both. Significant and coherent events (R + M+)	5/5	

Values reported are validations / events selected

**Fig. 4:**
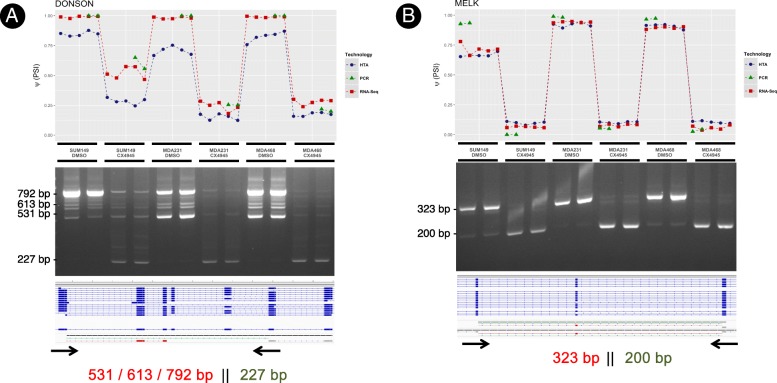
Estimated PSI (for RNA-seq, microarrays and PCR image analysis), PCR bands, the reference HTA transcriptome and the alternative paths of the *DONSON* (panel **a**) and *MELK* (panel **b**) genes in *R*^*+*^*M*^*+*^. Each of the points represents the same replicate in either of the three technologies. The last numbers shown are expected bands for the selected primers. If the number is shown to the left side of the double bars, the band corresponds to Path 1 of the event (long path). If shown to the right side, corresponds to Path 2 (short path)

Figure 4 shows the Ψ estimates and the PCR bands for two of the top-ranked events in R^+^M^+^ (gene names *DONSON* and *MELK*), with clear concordance of the splice index, Ψ, across the three technologies despite use of end-point (i.e. non-quantitative) PCR. Similar figures for events in the other AS categories are included in the additional material (Additional file 1: Figures S2 to S8).

### Statistics and Ψ for matched events are similar

**Fig. 5:**
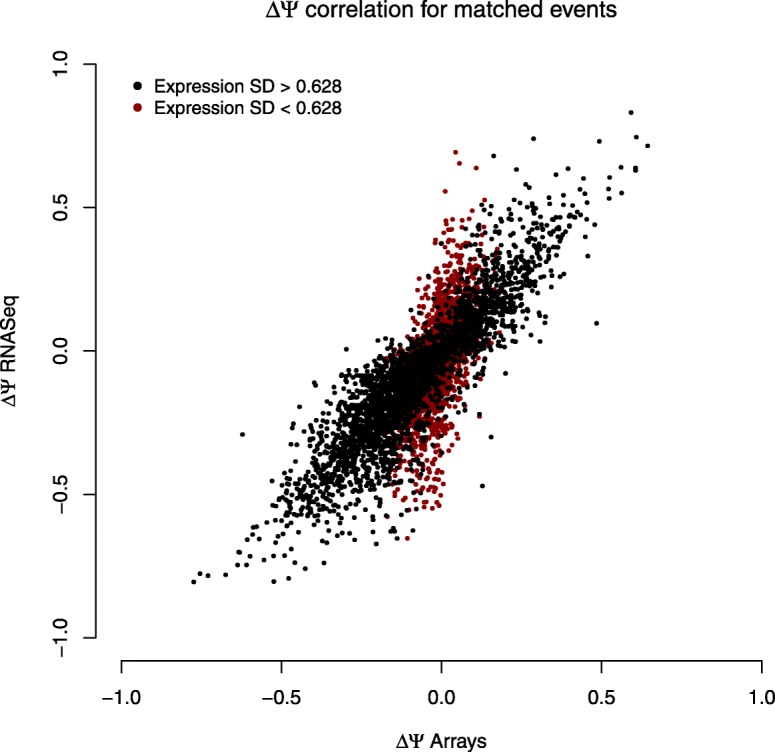
Increment of PSI for both microarrays and RNA-seq. The black (gray) dots represent events with high (low) standard deviation in the differential usage of the isoforms in both paths. Correlation between events with high and low variability are 0.90 and 0.61 respectively

Figure 5 shows the increment of the Ψ value estimated by EventPointer for events detected by both technologies. Correlation for AS events is over 0.90, and z-values of the statistical test are also similar (Additional file 1: Figure S1). PCR figures also show high coherence between the estimated Ψ using both technologies, especially for RNA-seq, and the PCR results (Fig. 4 and Additional file 1: Figure S2 to S8).

### Both technologies detect a similar distribution of AS types

**Fig. 6:**
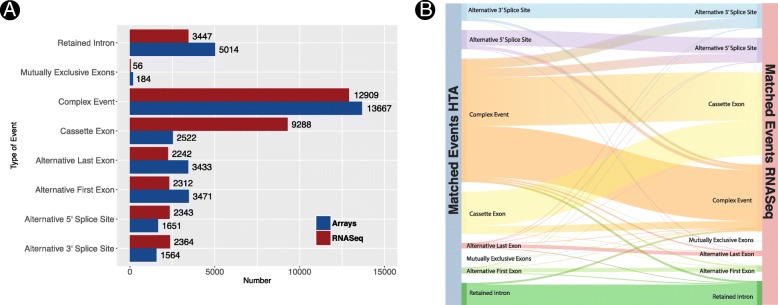
**a** Events detected using RNA-seq and array technologies. **b** Type of event after matching the events detected by both technologies

Figure 6a shows the number and type of AS events detected by the EventPointer algorithm on data from both profiling technologies. The number of detected cassette exons using arrays is smaller than that using sequencing (*p* value <1e-16, test for equality of proportions). In fact, after matching the events detected by both technologies, a large proportion of the cassette exons in RNA-seq appear as complex in microarrays (see Fig. 6b). The reason for this disparity is the complexity of the reference transcriptome used in the HTA array. For this analysis, we used the transcriptome provided by Affymetrix, which includes a range of annotation sources, e.g. RefSeq, Vega, Ensembl, MGC (v10), UCSC known genes and other sources for non-coding isoforms. The underlying transcriptome for HTA includes such a variety of isoforms that many detected AS events are labelled as complex.

In addition, the proportion of retained introns is smaller for RNA-seq (*p* value <1e-16, test for equality of proportions), perhaps owing to the coverage required to include a region as expressed by SGSeq (defaults to 0.5 FPKM) which may exclude weakly expressed introns.

### Power of arrays to detect events is approximately equivalent to shallow RNA-seq

The comparisons above suggest that that RNA-seq – at the depth of sequencing deployed here - detects a larger number of AS events at lower FDR.

We subsampled the initial RNA-seq data to 30% and 10% of the input, yielding approximately 30 million and 10 million reads respectively. Using these subsampled data, we estimated their FDR (see Table 4). Interpolating the FDR for them, the FDR using junction arrays is equivalent to the FDR of an RNA-seq experiment with sequencing depth of approximately 20 million reads.

**Table 4:** Obtained FDR values after subsampling the number of reads in the RNA-seq experiment

Decimation percentage	FDR
Decimated 10%	1.86e-3
Decimated 30%	6.97e-4

We hypothesize that the performance of the arrays could be greatly improved by removing bad-performing probesets. The RMA summarization algorithm withstands the presence of a few outlier probes. In fact, we have identified some probes that cross-hybridize in several loci of the transcriptome. However, if most of the probes that interrogate either of the paths or the reference do not perform well, the whole estimate of the splicing event will be compromised. Some of these cases can be detected since the signal of the events do not show internal coherence with the model (i.e. they show a large relative error if the weighted sum of the signals in Paths 1 and 2 and the reference Path are compared). These bad probesets are somehow expected: the design of junction probes has strong limitations since there is no room to select a probe with certain standards of quality (GC content, no cross-hybridization against the genome of the transcriptome, etc.). Owing to these probesets, a number of events are not being measured accurately. We have included in the additional material Additional file 1: Figure S9 to illustrate bad and good performing probesets: in panel A, it is shown an event with internal coherence and in panel B, an event with bad internal coherence. We have also included Additional file 1: Table S10 that shows the FDR for genes with large coherence (small relative error) and small coherence (large relative error). The FDR for genes with large internal coherence is 2 times bigger than for events with weak internal coherence.

The events that are not matched with RNA-seq are enriched in these pathological cases as shown in Table 2. On the contrary, the expected false discovery rate for matched events finds HTA arrays to be equivalent to RNA-seq with a depth of approximately 60 million reads. A proper filtering of the probes identifying events prone to errors could ideally take the arrays closer to the RNA-seq performance with this depth.

## Discussion

The main aim of this work was to quantitatively compare the performance of RNA-seq and junction array technologies to detect splicing events. To do this in a balanced manner, we adapted our algorithm EventPointer, originally developed for HTA arrays, to work also on RNA-seq data.

This study highlights:

- The creation of a real-world cell exposure dataset specifically relevant for the study of alternative splicing.
- Adaptation of an existing AS event detection algorithm to a cross-platform method to enable comparative application, and addition of percent splice index method.
- Strong correlation of splicing event detection in regions covered by both technologies, validated by PCR on a subset of top ranked events identified by both and each platform respectively.
- Benefits of RNA-seq in terms of coverage and flexibility, as expected, and higher validation rates in case of disagreement between technologies.
- Good performance of HTA arrays, estimated by approximation to be equivalent to relatively shallow RNA-seq in transcript regions covered.

Top-ranked events detected by each platform technology and estimates of relative event occurrence (ΔΨ) were validated by PCR. The relative occurrence estimates were also strongly correlated, close to 0.90 for events detected by both technologies. In addition to enabling comparison of the two profiling platforms, these results suggest also that the estimates themselves are a relevant addition to the original EventPointer algorithm.

We relied on SGSeq to build the splicing graph. Using other algorithms (such as Spladder) could impact the detected events especially for weakly expressed genes. According to a recent review of some of the authors [37], AltAnalyze is the only alternative that provides the analysis of splicing events both for arrays and RNA-seq. AltAnalyze characterizes each event by several values that must be integrated. For example, in a cassette exon, the signals of the probes in the exon, the flanking junctions and the skipping junction (and the equivalent coverage values for RNAseq) should be integrated to get a single figure of merit. We found it difficult to perform this integration since we are not the developers of this method. Nevertheless, using this method could be also informative to compare both platforms.

As might be expected in the absence of physical probe-sets, over 10,000 statistically significant events were identified by RNA-seq alone, the top ranked of which were validated via PCR. Approximately 3300 events were detected using microarrays but not detected using RNA-seq. In this case, some (3/5) of the top ranked events were validated and correspond to well-expressed genes. Those which did not may reflect the specific technical biases of each technology (cross hybridization of the probes, multi mapping reads, GC dependence, etc.)

A recent study [38] also compared RNAseq and arrays. This study was focused on patient derived samples instead of cell-lines as we did. This study pinpoints differences in the output between both technologies and states it in the title. In our case, the main divergence that we found between both technologies appears in the events that are not matched. Interestingly, the coherence between matched events -shown by Fig. 5 and the validated PCRs- is very strong: if an event is detected by both technologies, the increment of Ψ and its statistical significance (see Additional file 1: Figure S1) are very similar.

RNA-seq has inherent advantages over microarrays, including the ability to detect unlimited novel events. Furthermore, sensitivity can be improved by increasing sequencing depth. Another advantage of RNA-seq is its better approximation of gene/transcript concentrations (e.g. allowing to state a threshold based on the expression of an event). On the other hand, arrays were able to detect some weakly expressed events missed by RNA-seq and, in general across the comparisons, performed similarly to RNA-seq when treating well-expressed and well-defined transcriptional regions. As expected, a similar algorithmic approach applied to both platforms consumed less time and memory resources when treating microarray data than when treating RNA-seq data (See Table 5).

**Table 5:** Resources required for both technologies. Analysis was performed on 16 cores (Intel Xeon E5–2670 @ 2.60 GHz) with 64 GB of RAM Linux server running 64-bit CentOS distribution

	Computing time		Memory requirements		Storage requirements	
	*RNA-seq*	*HTA*	*RNA-seq*	*HTA*	*RNA-seq*	*HTA*
Mapping to the transcriptome (STAR)	11.5 h	–	32Gb	–	1023 GB	–
Splicing graph generation (SGSeq)	2 days (5 cores)	14 h	8 Gb per core	5 Gb	70.3 Mb	2 Gb
Event detection	7 min 16 s (10 cores)		1.2 Gb per core		643.6 Mb	
Statistical analysis	1 min 43 s	3 min 06 s	2 Gb	< 1Gb	6.2 Mb	11 Mb

## Conclusions

In conclusion, comparison of RNA-seq and junction microarrays using a cross-platform algorithm suggests that both technologies provide accurate identification of splice events. Moreover, predictions by both technologies tend to correlate strongly and yield similar results when compared by Ψ estimates and PCR. RNA-seq holds a clear advantage in terms of flexibility, and stronger PCR validation of events detected in one platform but not the other. As compared, HTA microarrays are shown nevertheless to provide a reasonable alternative to relatively shallow RNA-seq in the transcriptional regions that they reference.

## Methods

### Sample preparation

Triple negative breast cancer cell lines MDA-MB-231 and MDA-MB-468 were obtained from ATCC (Manassas, VA) with identification numbers HTB-26 and HT-132 respectively and SUM149 was purchased from Asterand plc (Detroit, MI). All cell lines were grown according to the suppliers’ recommendation. CK2 inhibitor CX-4945 (Selleckchem, Houston, TX) was dissolved in DMSO and stored frozen at − 80 °C until used.

To induce splicing events, cells were grown to ~ 70% confluence and treated with 1 μM CX-4945 or DMSO during 12 h in a total of 5 replicates per condition. Total RNAs were isolated using the RNeasy Mini Kit (Qiagen, Germantown, MD) according to the manufacturer’s protocol. Integrity of RNA was quantified using the Agilent 2100 Bioanalyzer (Agilent Biosystems, Foster City, CA). Samples were labeled and hybridized in Human Transcriptome arrays (HTA) by the Genomics Core Facility of the Center for Applied Medical Research (CIMA) following manufacturer’s instructions.

RNAseq was performed in the Center for Cooperative Research in Biosciences (CICBiogune) using the Illumina HiSeq2000 sequencing technology, HiSeq Flow Cell v3 and TruSeq SBS Kit v3. 2μg of RNA of each sample was sent for this purpose. The run type was strand specific, multiplexed with paired-end reads of 100 nucleotides each. The amount of RNA for hybridization and validation purposes was 5 μg.

STAR 2.4.0 h1 was used to align the reads against the human genome. The reference genome was Ensembl v.74, GRCh 37.75. The output were sorted BAM files. All the other parameters were set to the default values. The average sequencing depth was 49 million reads (9.8 billion nucleotides sequenced per sample).

The microarray data preprocessing was performed using the aroma.affymetrix framework using the standard RMA algorithm applied to probesets of the paths [30]. In addition, we used both platforms to quantify expression at the gene level. Results are shown in the Additional file 1. Gene expression was computed from RNA-seq data using Kallisto [14] to quantify expression using a pseudo-aligment method. Kallisto returns an estimate of the expression of all the isoforms for each gene. The overall expression of the gene was simply computed by summing up the expression of each the its isoforms. We estimated the expression of the arrays using the RMA algorithm in the aroma.affymetrix framework using a Brainarray reference file of the Ensembl 74 transcriptome.

### Event pointer for RNAseq

EventPointer is an R package to identify, classify and analyze alternative splicing events using microarrays and RNA-Seq data. The software is available for download at Bioconductor. A thorough description of EventPointer for microarrays can be found in [8]. This method has been extended to RNA-seq.

The concepts for detection, classification and statistical analysis are shared in EventPointer for the analysis of both technologies. The main difference of EventPointer for RNA-seq compared with that of microarrays are the ones associated with the type of input data (CEL or BAM files). The R code for the analysis is available at https://github.com/jpromeror/SplicingComparison.

EventPointer requires a splicing graph -a directed graph used to represent the structure of the different isoforms of a given gene [39] - as input to detect splicing events. EventPointer for RNA-seq uses SGSeq [24] to build the corresponding splicing graphs from BAM files. The complexity of the splicing graph can be controlled in SGSeq by setting different thresholds in the expression values of splicing junctions of the splicing graph (by default set to 2 FPKM). For RNA-seq, the splicing graphs are constructed for every single experiment. On the contrary, in the case of microarrays the same splicing graph (and the corresponding CDF) is used for all the experiments run on the same type of microarray (HTA or, more recently Clariom-D).

The input data for the statistical analysis is different in both technologies: signal values of the probes in microarrays and counts in RNA-seq. In order to deal with reads, Voom [17] is applied to preprocess the RNA-seq count data. The statistics to deal with the processed RNA-seq data is identical to the one used for microarray data and hence, the same statistical tests -based on limma [33]- are applied to both technologies.

As output, EventPointer provides a table with the following information associated to each detected alternatively spliced event: gene identifier, genomic position, type of event, statistical parameters and ΔΨ values. Additionally, EventPointer generates a “Gene Transfer Format” (GTF) file that can be used with the Integrative Genomics Viewer (IGV) [36] to view the structures of each detected alternative splicing event. This visualization facilitates the interpretation of the detected events and the design of primers for the validation of the events using standard PCR.

### Estimation of PSI

We have included a novel algorithm to estimate Ψ that can be applied to both RNA-seq and microarrays. Assuming that the signal of a probe-set in microarrays and the number of reads within a region of the transcriptome in RNA-Seq depend on the product of an affinity value of the probe-set (or the equivalent length in RNA-seq) and the concentration of the interrogated isoforms in the paths, the following equation holds

1 $$ {S}_i={a}_i\bullet {t}_i $$

where *S*_*i*_ is the measured expression value of path *i*, *a*_*i*_ is the affinity of the probes or equivalent length of the path *i* and *t*_*i*_ is the concentration of the isoforms mapped to path *i*. The affinity values (or equivalent lengths) and concentration values are assumed to be unknown and must be estimated from the data.

Particularizing the above equation to each of the paths and taking into account that the concentration of the isoforms in the reference path must be the sum of those of paths 1 and 2, the following equations are obtained:

2 $$ {S}_1={a}_1\bullet {t}_1 $$

3 $$ {S}_2={a}_2\bullet {t}_2 $$

4 $$ {S}_R={a}_R\bullet {t}_R={a}_R\left({t}_1+{t}_2\right) $$

In turn, the signal value of the reference path can be expressed as the sum of the signal values of paths 1 and 2 as follows,

5 $$ {S}_R={a}_R{a}_1^{-1}{S}_1+{a}_R{a}_2^{-1}{S}_2=u{S}_1+v{S}_2 $$

where *u* and *v* represent the fraction of the affinities of the mapped probe-set (or equivalent lengths) in the reference path and paths 1 or 2 respectively. The values of *u* and *v* can be estimated from signal data.

Dividing eq. (2) with eq. (4) we get,

6 $$ \frac{S_1}{S_R}=\frac{a_1{t}_1}{a_R\left({t}_1+{t}_2\right)} $$

Combining eqs. (5) and (6), the desired equation of the Percent Spliced Index (Ψ) used in EventPointer is obtained:

7 $$ \varPsi =\frac{t_1}{t_1+{t}_2}=\frac{u{S}_1}{S_R}=\frac{u{S}_1}{u{S}_1+v{S}_2} $$

Note that Ψ can be directly obtained from signal values once *u* and *v* are known. This equation does not require the estimation of the affinities (difficult to predict accurately) to compute Ψ. On the contrary, it simply requires to estimate *u* and *v* from signal values using eq. (5). In the case of RNA-seq, the equivalent lengths are known a priori and hence *u* and *v*. However, using this approach has an advantage: the estimates of these lengths can accommodate the potential lack of uniformity of the reads.

Note that *u* and *v* must be positive, similar between them and close to one. The first affirmation is trivial since affinity values (or equivalent lengths) are always positive. In microarrays, probe-sets are composed by several probes and their overall affinity are expected to be similar to each other, since these affinities are a median of the average of the affinities of the probes that build up them. Therefore *a*_1_ ≈ *a*_2_ ≈ *a*_*R*_,and *u* ≈ *v* ≈ 1. A similar reasoning can be applied to RNA-seq, if using coverage instead of read counts, since the coverage of the reference path is expected to be close to the sum of the coverages of paths 1 and 2.

These two fractions can be estimated from eq. (5) by using non-negative least squares as follows:

8 $$ {\displaystyle \begin{array}{c}\min \left\Vert Ax-b\left\Vert {}_2\right.\right.\\ {}s.t.x\ge 0,x\in {R}^2,A\in {R}^{m\;x\;n}\end{array}} $$

where,

9 $$ A=\left[\begin{array}{cc}\boldsymbol{Signal}\ \boldsymbol{P}\mathbf{1}& \boldsymbol{Signal}\ \boldsymbol{P}\mathbf{2}\\ {}\lambda & -\lambda \\ {}\lambda & 0\\ {}0& \lambda \end{array}\right];x=\left[\begin{array}{c}u\\ {}v\end{array}\right];b=\left[\begin{array}{c}\boldsymbol{Signal}\ \boldsymbol{R}\\ {}0\\ {}\lambda \\ {}\lambda \end{array}\right] $$

The penalty factor λ is added to force the equation to fulfill the previous considerations: *u* and *v* must be similar and close to 1. In our results, we found that the estimates were not sensitive to the specific value of λ if there is differential alternative splicing. If the relative usage of both paths is similar and therefore, Ψ is constant, the results are more sensitive to the value of λ. This fact is shown in Fig. 4: the correlation is much better for events that show variability in the relative expression of both paths.

The residuals of this model can be used to test if the additive model of eqs. 2, 3 and 4 holds. We computed the relative error of the residuals as follows:

$$ \upvarepsilon =\frac{{\left\Vert \left(u\cdotp \boldsymbol{Signal}\ \boldsymbol{P}\mathbf{1}+v\cdotp \boldsymbol{Signal}\ \boldsymbol{P}\mathbf{2}\right)-\boldsymbol{Signal}\ \boldsymbol{R}\right\Vert}_2}{{\left\Vert \boldsymbol{Signal}\ \boldsymbol{R}\right\Vert}_2} $$

If the relative error is large, the additive model does not fit the data and, therefore, the estimates are expected to be less reliable. In order to test this, we divided the events according to the relative error. The events with top 50% relative error have FDR two times larger than the bottom 50% as shown in Additional file 1: Table S10.

### Statistical analysis

The comparison and analysis of the profiling data was done using a linear model. The design matrix was built considering both the cell line and treatment with CX4945 as factors. The interaction between cell line type and treatment was not considered.

The selected contrasts test for the difference between control samples (DMSO) and drug exposed ones (CX4945) controlling for the cell-type. The complete experimental design in the form of design and contrast matrices is included in Additional file 1: Table S9.

EventPointer includes several statistical methods to state the significance of an event. In this experiment, the events are considered to be statistically significant if there is a change in the expression of the isoforms associated to each of the alternative paths, this change occurs in opposite direction, i.e. opposite signs for the fold changes and the summarized p.value is significant (*p* value< 0.001).

In order to compare the arrays with different sequencing depths, we subsampled the RNAseq data to 30 and 10 million reads and rerun the whole pipeline with these data. The FDR for 30 million reads was better than using arrays. On the contrary, using 10 million reads the FDR was worse than using arrays. Interpolating both data, the FDR for arrays is similar to a depth of 20 million reads.

### Filters used to include the events

For arrays, the signal of the probe-sets interrogating each of the alternative paths involved in a splicing event, must be expressed more than a certain threshold in at least one sample. This threshold is the 25% quantile of the expression of the signal in the reference paths for all the events included in the array. For RNAseq, the edges of the splicing graph (junction reads) are included only if their expression is at least 2 FPKM in at least one sample (SGSeq defaults).

### Matching of the events using different technologies

Let’s assume that *A*_*R*_ and *A*_*M*_ are, possibly non-contiguous, regions of the genome that correspond to path A using either technology (*A*_*R*_ for RNA-seq and *A*_*M*_ for HTA). *B*_*R*_ and *B*_*M*_ have a similar description for path B and *R*_*R*_ and *R*_*M*_ for the reference path in each technology. Two events are considered to match if any of the following two expressions is true:

10 $$ \left(\left({A}_R\subset {A}_M\right)|\left({A}_M\subset {A}_R\right)\right)\&\left(\left({B}_R\subset {B}_M\right)|\left({B}_M\subset {B}_R\right)\right)\&\left(\left({R}_R\cap {R}_M\right)\ne \varnothing \right) $$

11 $$ \left(\left({A}_R\subset {B}_M\right)|\left({B}_M\subset {A}_R\right)\right)\&\left(\left({B}_R\subset {A}_M\right)|\left({A}_M\subset {B}_R\right)\right)\&\left(\left({R}_R\cap {R}_M\right)\ne \varnothing \right) $$

In these expressions, (*x* ⊂ *y*) is true if the genomic region *x* is a subset of the genomic region *y* (the nucleotide sequence of *x* is a substring of the nucleotide sequence in *y*). Besides, the operators “|” and “&” and the logical OR and AND operations. If (*x* ⊂ *y*)|(*y* ⊂ *x*), then one of the regions is contained in the other are considered to be “compatible”. On the other hand, (*x* ∩ *y*) ≠ ∅ means that regions x and y overlap in the genome. Therefore, the first expression is true if both paths *A*_*R*_ and *A*_*H*_ are compatible, *B*_*R*_ and *B*_*M*_ are compatible and *R*_*R*_and *R*_*M*_ overlap. The second expression is true if path *A*_*R*_ and path *B*_*M*_ are compatible and also path *A*_*M*_ and *B*_*R*_ are compatible and, again, and *R*_*R*_ and *R*_*M*_ overlap.

Within an event, the longer path in the transcriptome is assigned the name “A” and the other the B. The second eq. (11) takes into account that, in some few cases, the name of the paths can be switched in both technologies.

### PCR validation

For each splicing event, an end-point PCR was run using primers designed in the exons that flank the event of interest. RNA was retro-transcribed and the PCR was performed an analyzed as previously described [40]. Primers used are shown in Additional file 2.

## Additional files

Additional file 1: Vignette on the comparison based on expression analysis. **Figure S1** to **S9**. Experiment design and contrast matrices. **Table S10**. (PDF 7766 kb)

Additional file 2: Excel file with characteristics of the validated events and PCR primers. (XLSX 27 kb)

Additional file 3: Compressed file including the GTF files generated for the matched events. (ZIP 15 kb)

## Abbreviations

AS: Alternative Splicing
CDF: Chip Definition File
FDR: False Discovery Rate
GTF: Gene Transfer Format
HTA: Human Transcriptome Array
NGS: Next-Generation Sequencing
PCR: Polymerase Chain Reaction
TNBC: Triple-Negative Breast Cancer

## Footnotes

1 Average sequencing depth for RNA-seq was approx. 98 million (paired-end, stranded protocol), yielding on average approx. 49 million fragments per sample.
