# Supplementary material for: Comparison of RNA-seq and microarray platforms for splice event detection using a cross-platform algorithm
Source: BMC Genomics. 2018 Sep 25;19:703. doi: 10.1186/s12864-018-5082-2 (PMC6156849; doi:10.1186/s12864-018-5082-2)
Supplement: Supplementary file 1 — Vignette on the comparison based on expression analysis. Figure S1 to S9. Experiment design and contrast matrices. Table S10. (PDF 7766 kb) [file 12864_2018_5082_MOESM1_ESM.pdf]

Comparison of gene expression profiles between RNA-Seq and Microarray

Juan P. Romero, F. Carazo and Angel Rubio

July 03, 2018

The main objective of this section is to compare RNAseq and Microarrays at the gene level. Although this issue has been already done in the past, we consider important to double-check it for our data as a first step in our study. As it shown below, correlation between technologies for expressed genes is around 0.75.

Content

1. Introduction
2. Biological replicates
3. Correlation of expression values
4. Effect of gene expression levels
5. Individual correlations
6. Conclusions

1. Introducction

The expression profiles of 21,248 common genes were compared between the two platforms. In both cases, it has been used the same version of the Ensembl Transcriptome (Ensembl GRCh 37.74).

- **RNAseq** The average sequencing depth for the RNAseq was 98 million (paired-end, single stranded protocol). The expression has been calculated using Kallisto and summarizing it by genes.

- **Microarray** Samples were hybridizated using the HTAv2 platform of Affymetrix and following the standard pathway of Aroma Project http://www.aroma-project.org/. Then it has been performed RMA analysis using the CDF provided by Brainarray (Version 18) http://brainarray.mbni.med.umich.edu/.

Samples from three different cell lines (SUM149, MDA231 and MDA468). Half of the samples were treated using CX4945 and the other half using DMSO (control). 5 replicates for each cell line in both procedures. A total of 30 samples:

- Cell line: MDA231 Treatment: CX4945 -> 5 replicates
- Cell line: MDA231 Treatment: DMSO -> 5 replicates
- Cell line: MDA468 Treatment: CX4945 -> 5 replicates

- Cell line: MDA468 Treatment: DMSO -> 5 replicates
- Cell line: SUM149 Treatment: CX4945 -> 5 replicates
- Cell line: SUM149 Treatment: DMSO -> 5 replicates

2. Biological replicates

The correlation coefficients between biological replicates range from 0.988 to 0.996 in microarray, and the corresponding correlation coefficients are 0.996 to 0.997 in RNA-Seq. The associated p-values with sample size of 21,248 genes are less than 2e-16 in both cases. Note that the correlation was calculated using log2 transformed expression values.

As it is shown in the following figures, RNA-Seq has a better correlation than microarray. For low expressed genes, variability is higher in RNA-Seq, specially for genes whose expression levels are near to zero.

Here it is represented the relation of the expression between two replicates of RNAseq (*RNAseq Rep 1* and *RNAseq Rep 2*), and, analogously for HTA (*HTA Rep 1* and *HTA Rep 2*). We also show the values of both linear regression. We just show one plot for each technology because results are similar for all samples.

```
# The correlation of gene expression for biological replicates in RNA-Seq
.
```

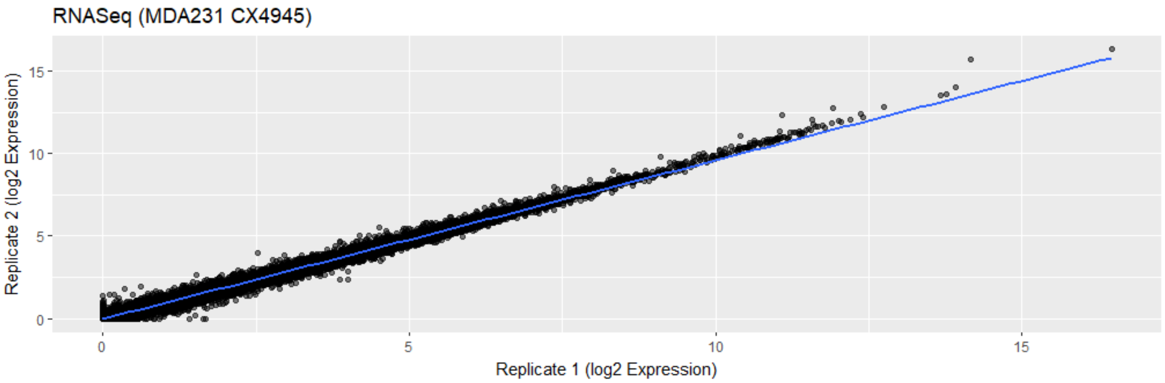


```
##
## Call:
## lm(formula = log2(RNAseq[, 1] + 1) ~ log2(RNAseq[, 2] + 1))
##
## Residuals:
##      Min       1Q   Median       3Q      Max
## -2.08399 -0.08578 -0.03209  0.11462  1.61697
##
## Coefficients:
##                        Estimate Std. Error t value Pr(>|t|)
## (Intercept)           0.0545180  0.0019715   27.65   <2e-16 ***
## log2(RNAseq[, 2] + 1) 1.0305862  0.0006062 1700.09   <2e-16 ***
```

```
## ---
## Signif. codes:  0 '***' 0.001 '**' 0.01 '*' 0.05 '.' 0.1 ' ' 1
##
## Residual standard error: 0.2052 on 21246 degrees of freedom
## Multiple R-squared:  0.9927, Adjusted R-squared:  0.9927
## F-statistic: 2.89e+06 on 1 and 21246 DF,  p-value: < 2.2e-16

# The correlation of gene expression for biological replicates in HTAv2.
```

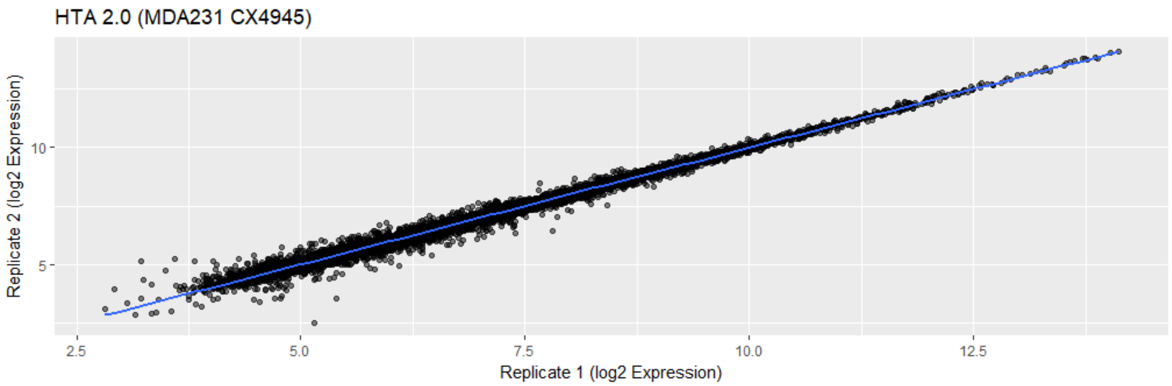


```
##
## Call:
## lm(formula = log2(HTA[, 1] + 1) ~ log2(HTA[, 2] + 1))
##
## Residuals:
##      Min       1Q   Median       3Q      Max
## -1.92464 -0.05620 -0.00089  0.05412  2.60136
##
## Coefficients:
##                     Estimate Std. Error t value Pr(>|t|)
## (Intercept)        0.0275815  0.0044414    6.21 5.39e-10 ***
## log2(HTA[, 2] + 1) 0.9961916  0.0006203 1606.02  < 2e-16 ***
## ---
## Signif. codes:  0 '***' 0.001 '**' 0.01 '*' 0.05 '.' 0.1 ' ' 1
##
## Residual standard error: 0.1272 on 21246 degrees of freedom
## Multiple R-squared:  0.9918, Adjusted R-squared:  0.9918
## F-statistic: 2.579e+06 on 1 and 21246 DF,  p-value: < 2.2e-16
```

3. Correlation of expression values

If the gene expression is stored as a rectangular matrix of genes x samples, the median correlation of gene expression between both technologies taking row or column vectors is 0.68 and 0.5 respectively.

Since the affinities of the probes are different for different genes, the average correlation by columns is much smaller than by rows. In other words, the signal that provides the expression of two different genes may be different either because they

have different expression or because the affinities of the probes interrogating both genes are different. It is not advisable to compare the signal of two genes within the same sample using the microarray technology.

Below it is shown the density graph of correlations (taking rows) between RNAseq and Microarrays (HTAv2).

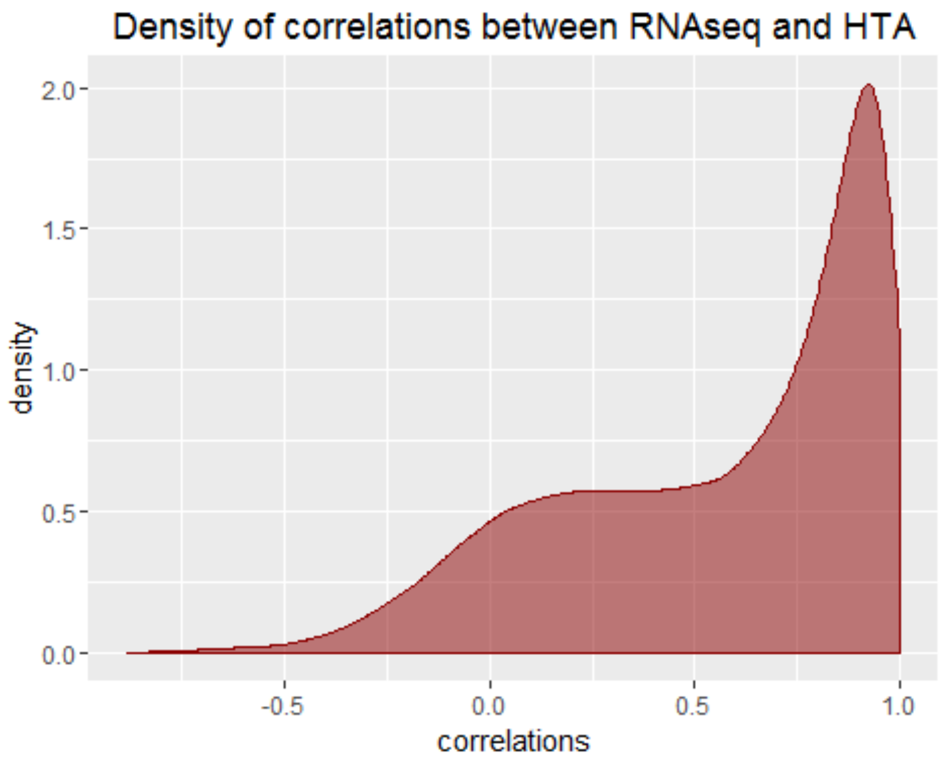

As it can be seen in the following figure, the correlations strongly rely on the biotype's gene and protein coding genes are those which have greater correlations.

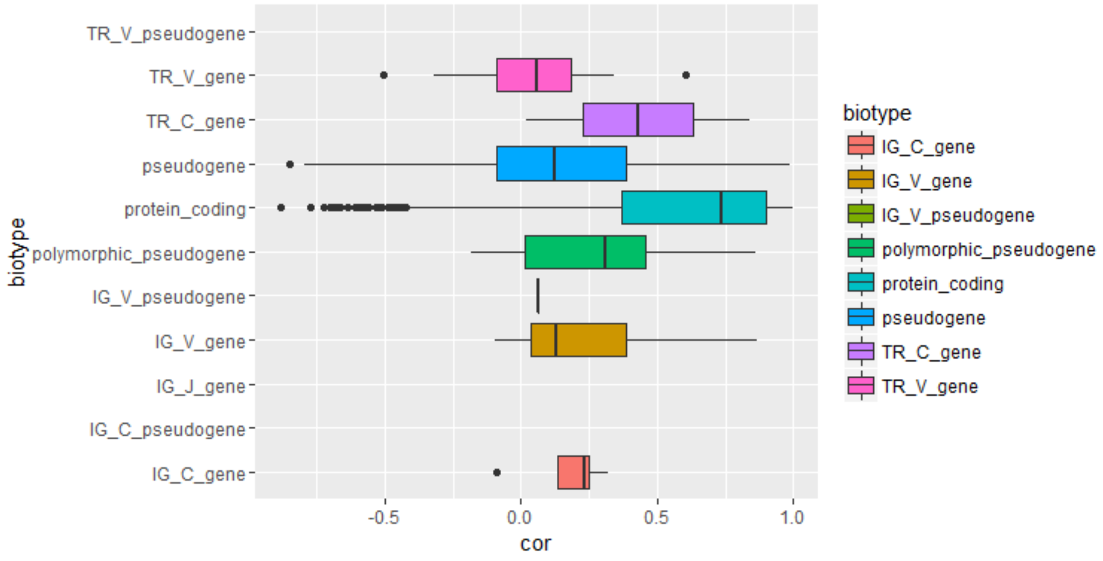

Protein coding genes are the most frequent genes in our data. Hereinafter we will compare protein coding genes.

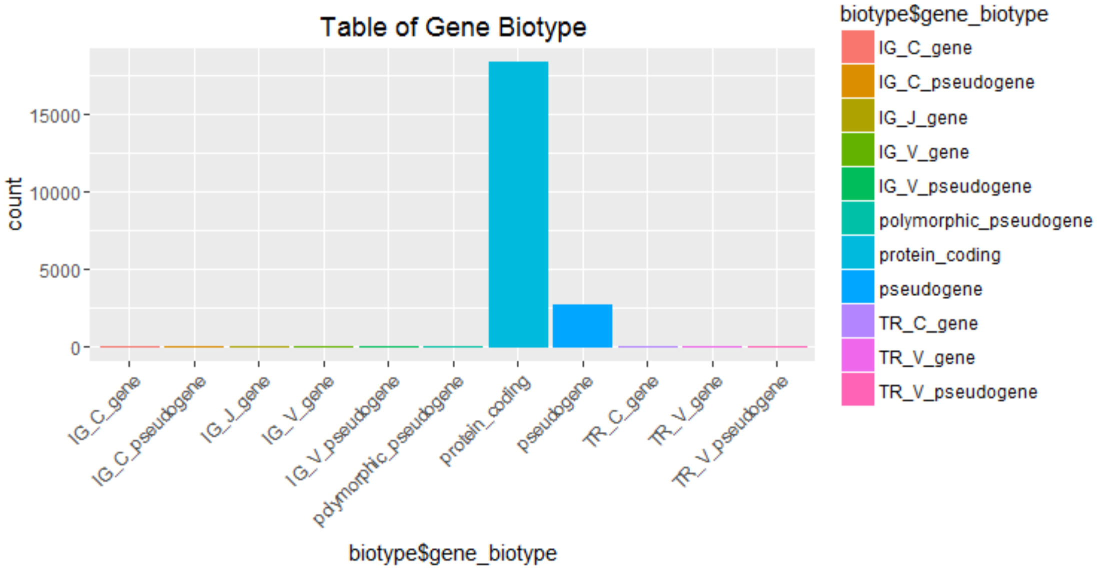

4. Effect of gene expression levels

In addition to the gene biotype, the levels of gene expression are also expected to affect correlations. Below it can be seen the boxplot of correlations between RNAseq and HTA grouped by quantiles of expression in each technology. The overall variance of correlation was much broader in HTA than that in RNAseq, especially at the lower end.

Note the relative expression level of each gene in the last plot was determined based on the median of log2 transformed expression values in all 30 samples for each gene.

The median of correlations of the 50% high expressed genes is 0.75 (by rows) and 0.73 (by columns), it can be remembered from the first section that this values were 0.68 and 0.5 for rows and columns respectively when we consider all genes.

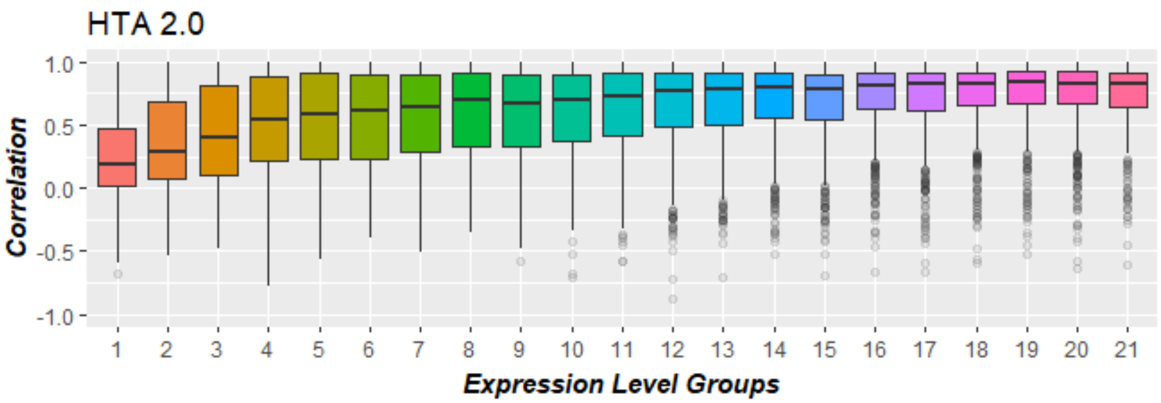

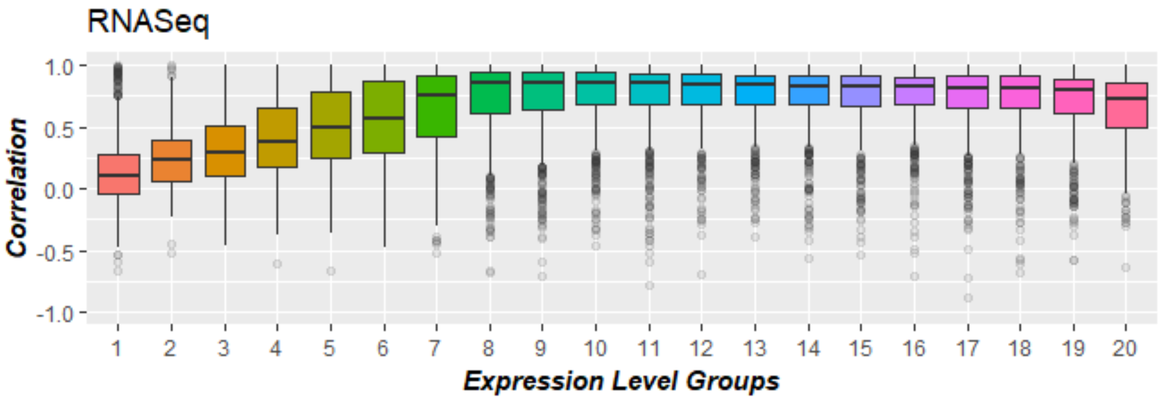

5. Individual correlations

Finally, we represented the correlations between single samples using: 1) only RNAseq, 2) only HTA, and 3) both technologies.

The correlation between samples of same Cell Lines (shown with same colors dark and light) are up to 0.95 in all cases. The accuracy of RNAseq is large better than in arrays as it is shown in figures 1) and 2). We can also seen that sample *MDA468_DMSO_1* in HTA, has low correlations with other ones maybe because any problem in hybridization.

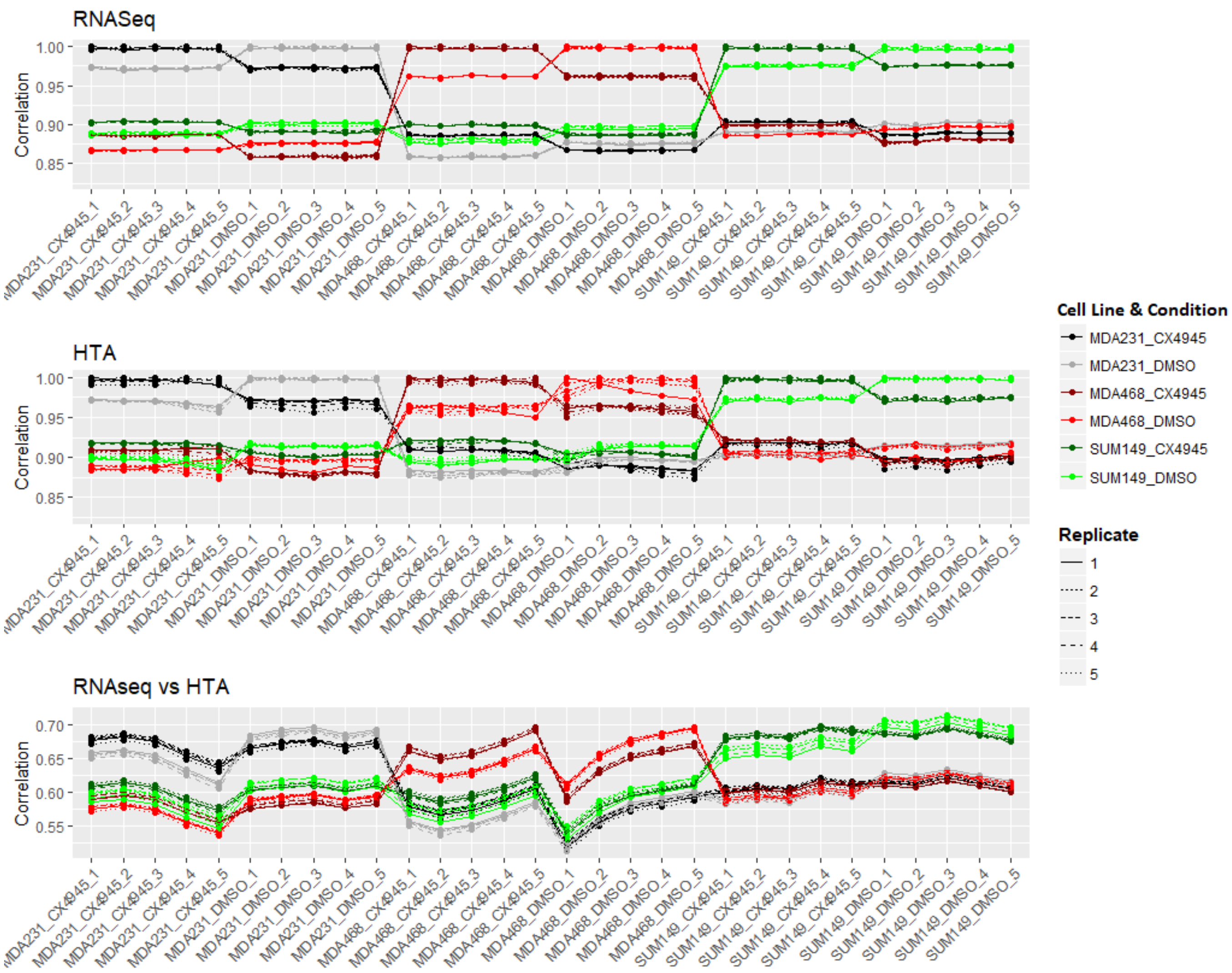

6. Conclusions

The expression profiles of 21,248 common genes were compared between the two platforms. We can confirm that both technologies -RNAseq and Microarrays- are given coherent information of gene expression profiles with a median of correlations between platform of 0.75. RNAseq with this depth of coverage is more reliable than HTA.

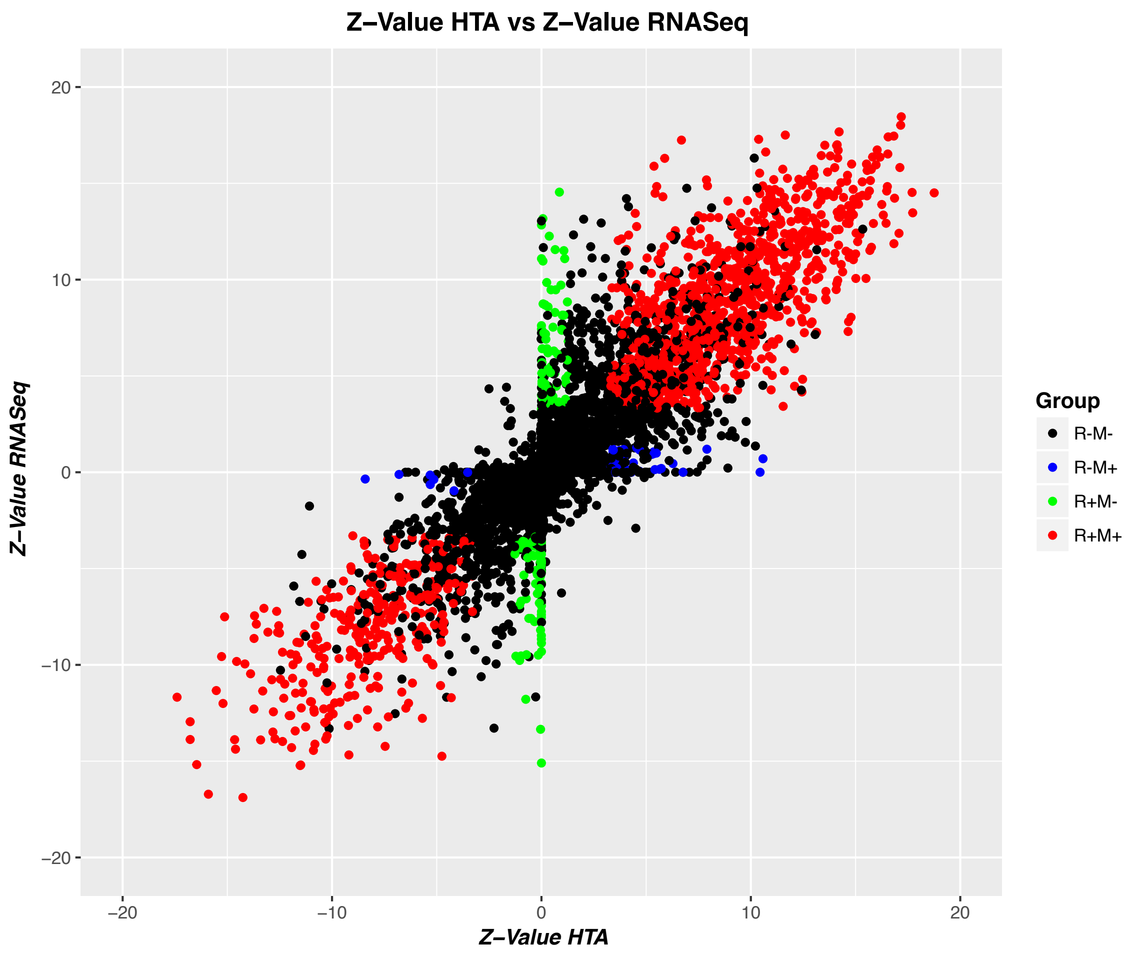

Figure S1. Scatterplot of the splicing z-value provided by EventPointer for RNASeq and HTA 2.0. The colors represent the groups: R-M- (black), R-M+ (blue), R+M- (green) and R+M+ (red). The correlation is 0.89.

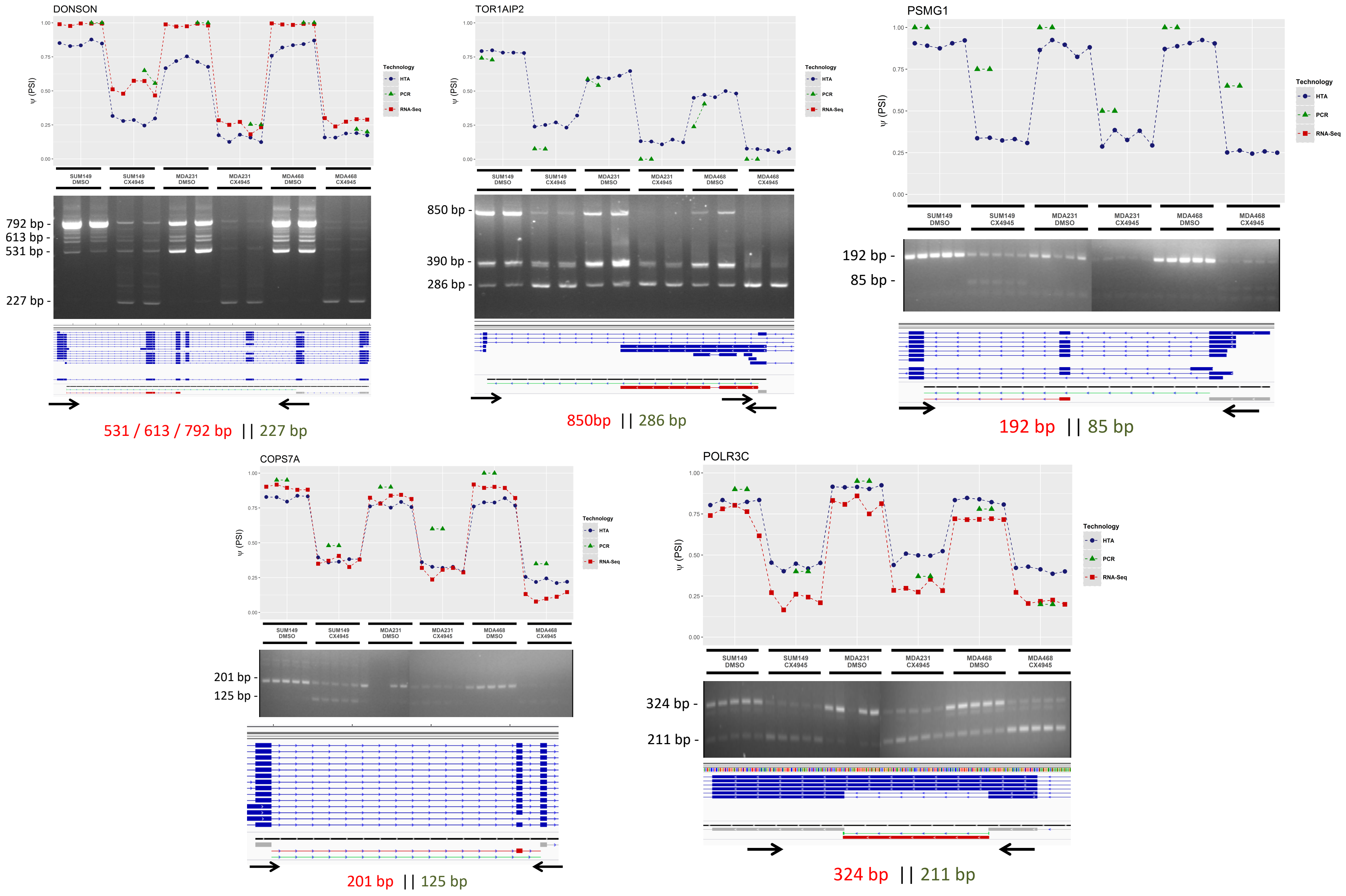

Figure S2. Estimated PSI, PCR bands, the reference HTA transcriptome and the alternative paths of the *DONSON*, *TOR1AIP2*, *PSMG1*, *COPS7A* and *POLR3C*. These genes correspond to the top 5 predicted events by EventPointer using Affymetrix HTA 2.0. All of them were considered validated.

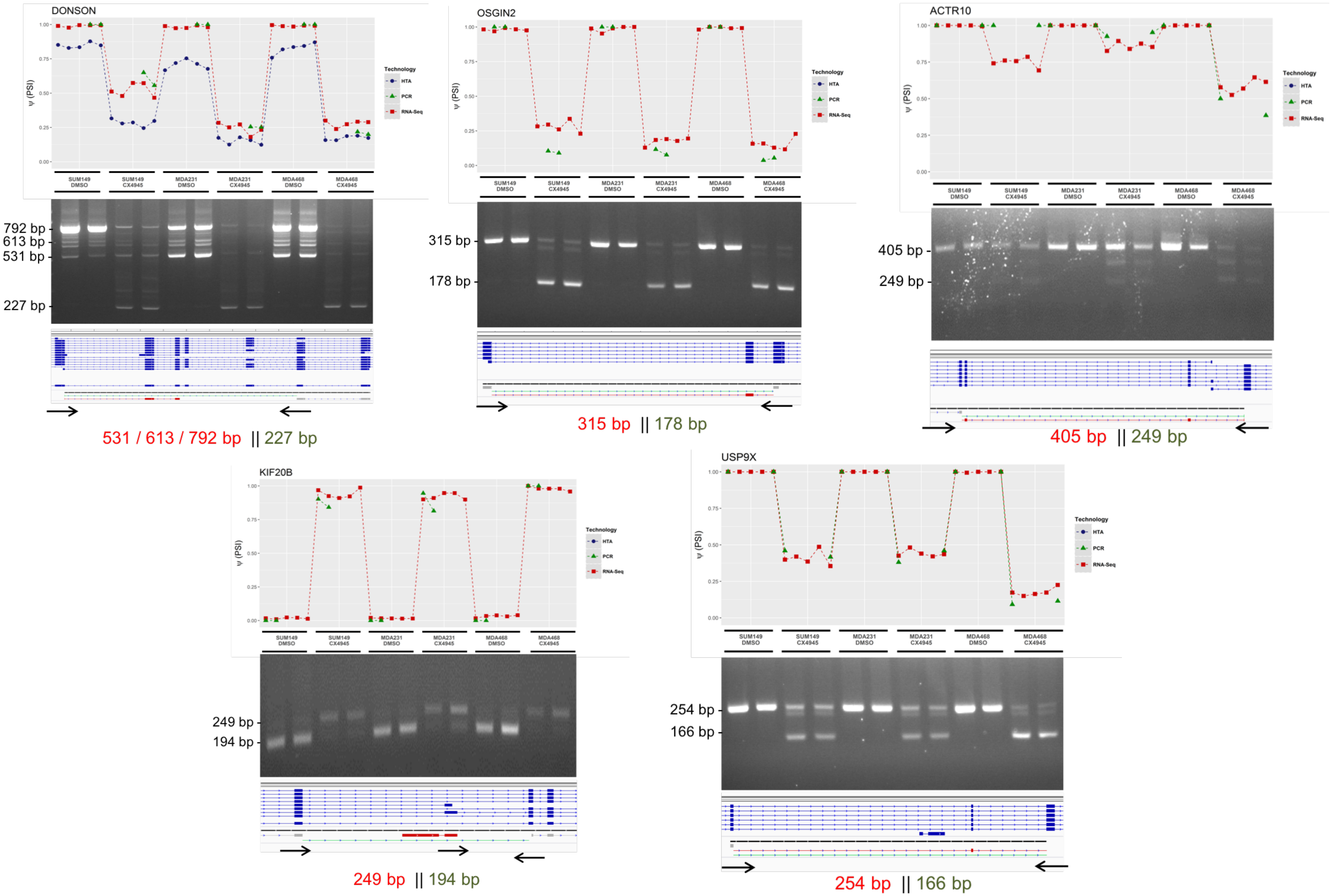

Figure S3. Estimated PSI, PCR bands, the reference HTA transcriptome and the alternative paths of the *DONSON*, *OSGIN2*, *ACTR10*, *KIF20B* and *USP9X*. These genes correspond to the top 5 predicted events by EventPointer using RNASeq. All of them were considered validated.

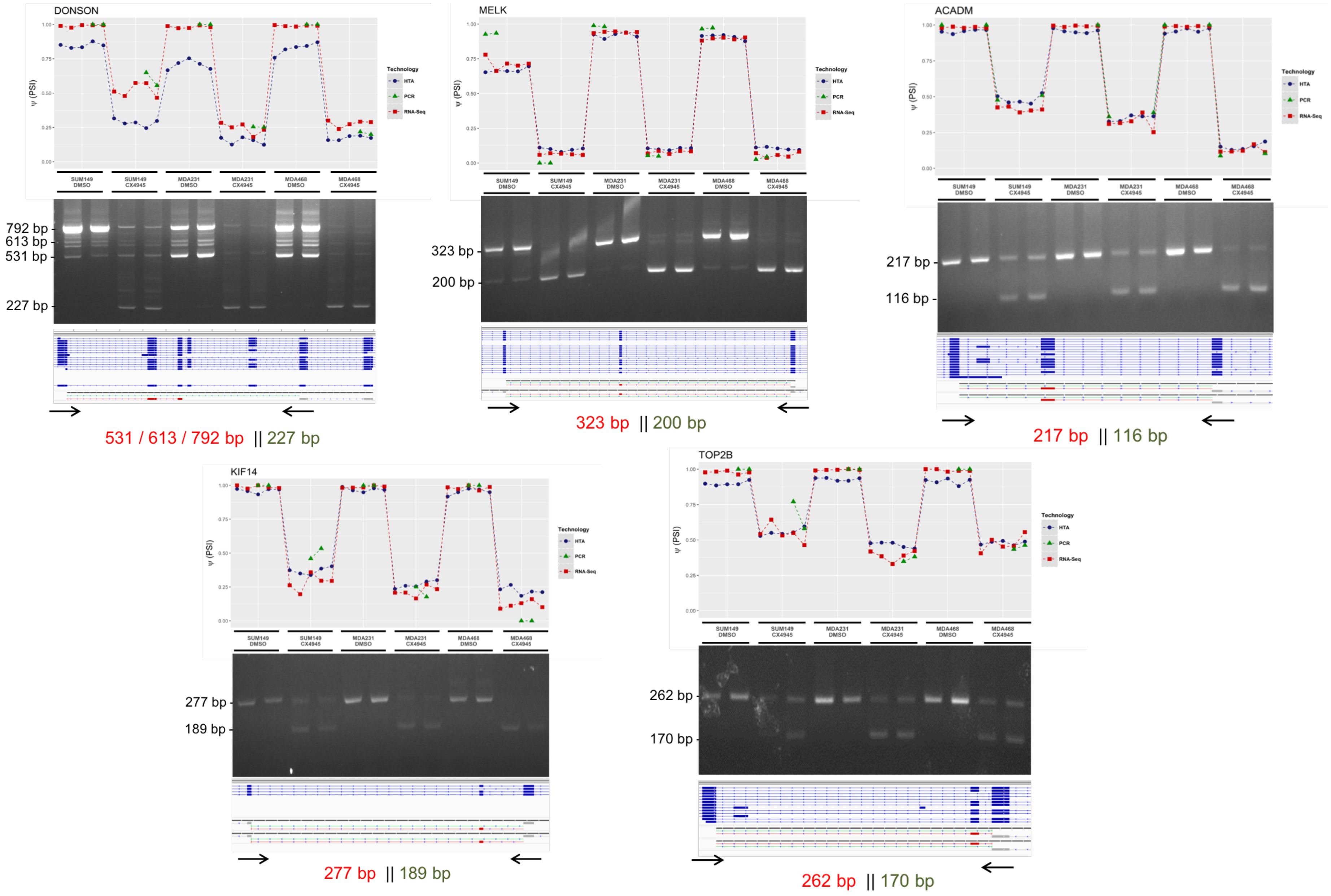

Figure S4. Estimated PSI (for RNA-seq –red-, microarrays –blue- and PCR image analysis –green-), PCR bands, the reference HTA transcriptome and the alternative paths of the *DONSON*, *MELK, ACADM, KIF14 and TOP2B*. These genes were found to be significant by both technologies. All of them were considered validated. The arrows below the alternative paths depict the location of the primers and the numbers shown are the expected lengths of the PCR bands with the selected primers.

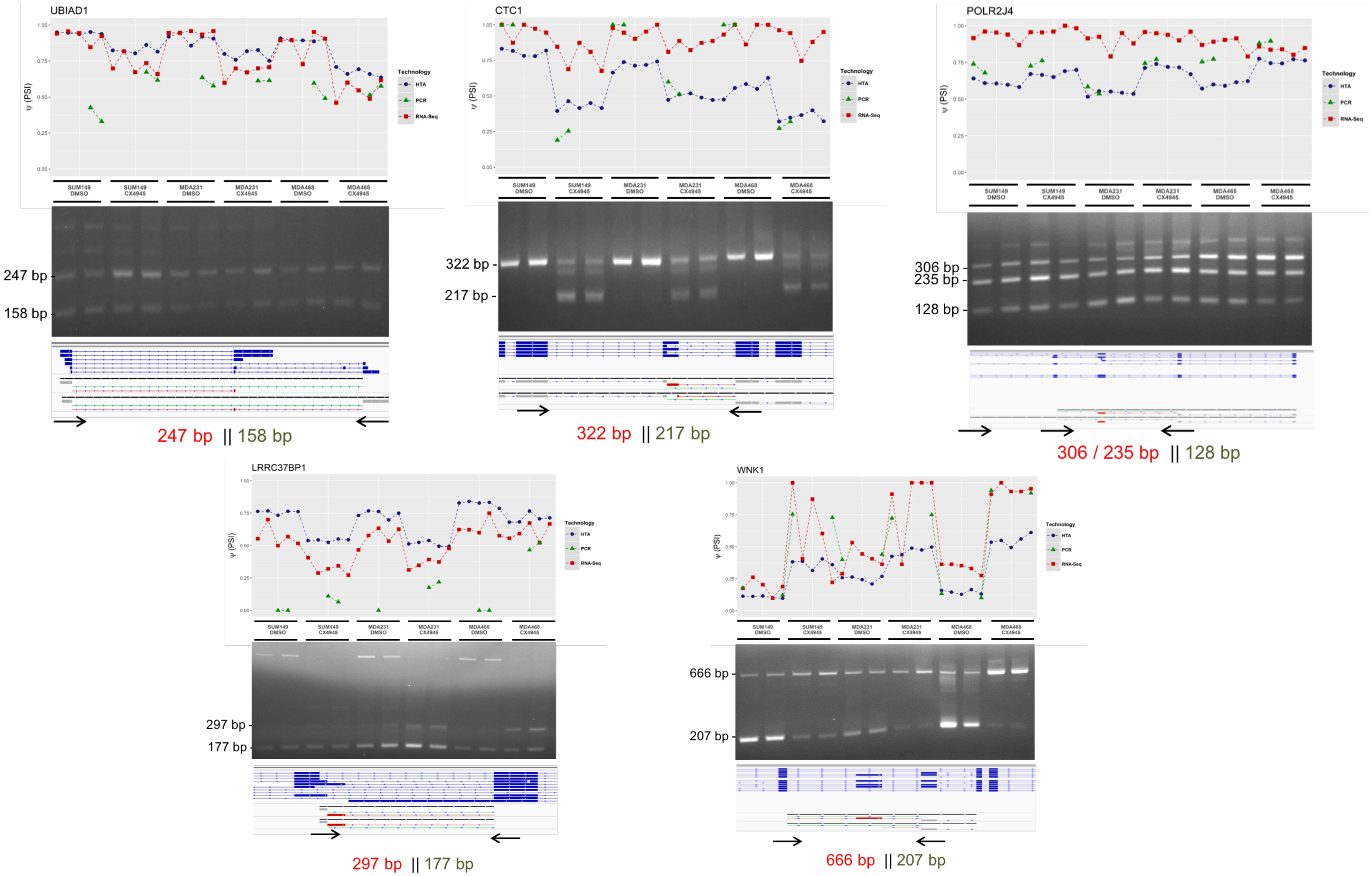

Figure S5. Estimated PSI (RNA-seq –red-, microarrays –blue- and PCR image analysis –green-), PCR bands, the reference HTA transcriptome and the alternative paths of the *UBIAD1*, *CTC1*, *POLR2J4*, *LRRC37BP1* and *WNK1* genes. These genes detected by both technologies and found to be significant only by HTA. *UBIAD1* and *POLR2J4* bands does not distinguish treatment and control as clearly as other samples. In two of the genes (*UBIAD1*, *LRRC37BP1*) unpredicted long bands (probably intron retentions) appeared in the PCRs. *CTC1*, *LRRC37BP1* and *WNK1* were considered to be validated.

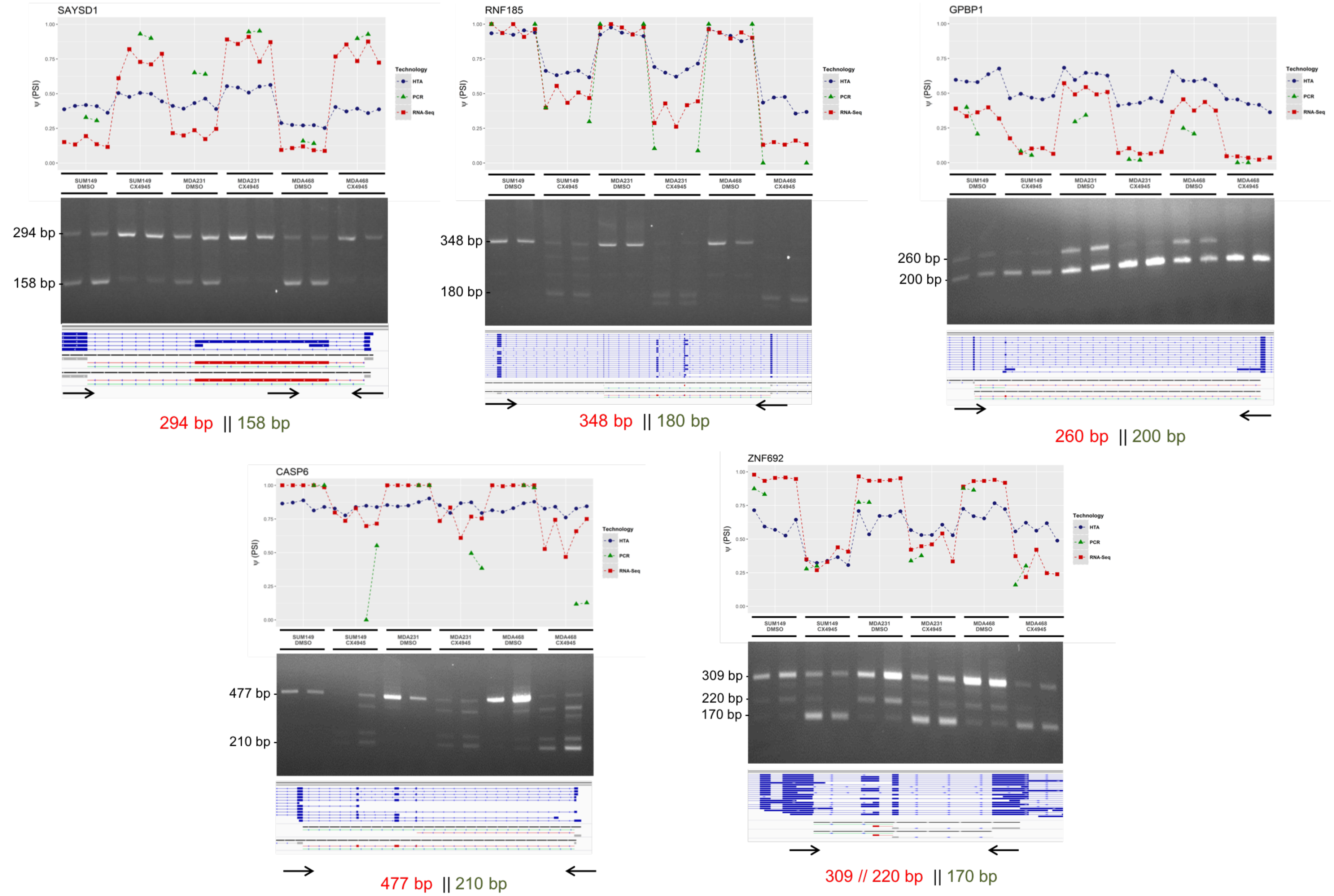

Figure S6. Estimated PSI (for RNA-seq –red-, microarrays –blue- and PCR image analysis –green-), PCR bands, the reference HTA transcriptome and the alternative paths of the *SAYSD1*, *RNF185*, *GPBP1*, *CASP6* and *ZNF692* genes. These genes were found to be significant only by RNA-seq but were detected also by HTA. All of them were considered validated (i.e. the prediction by RNA-seq is correct). CASP6 includes some additional bands non-detected by EventPointer.

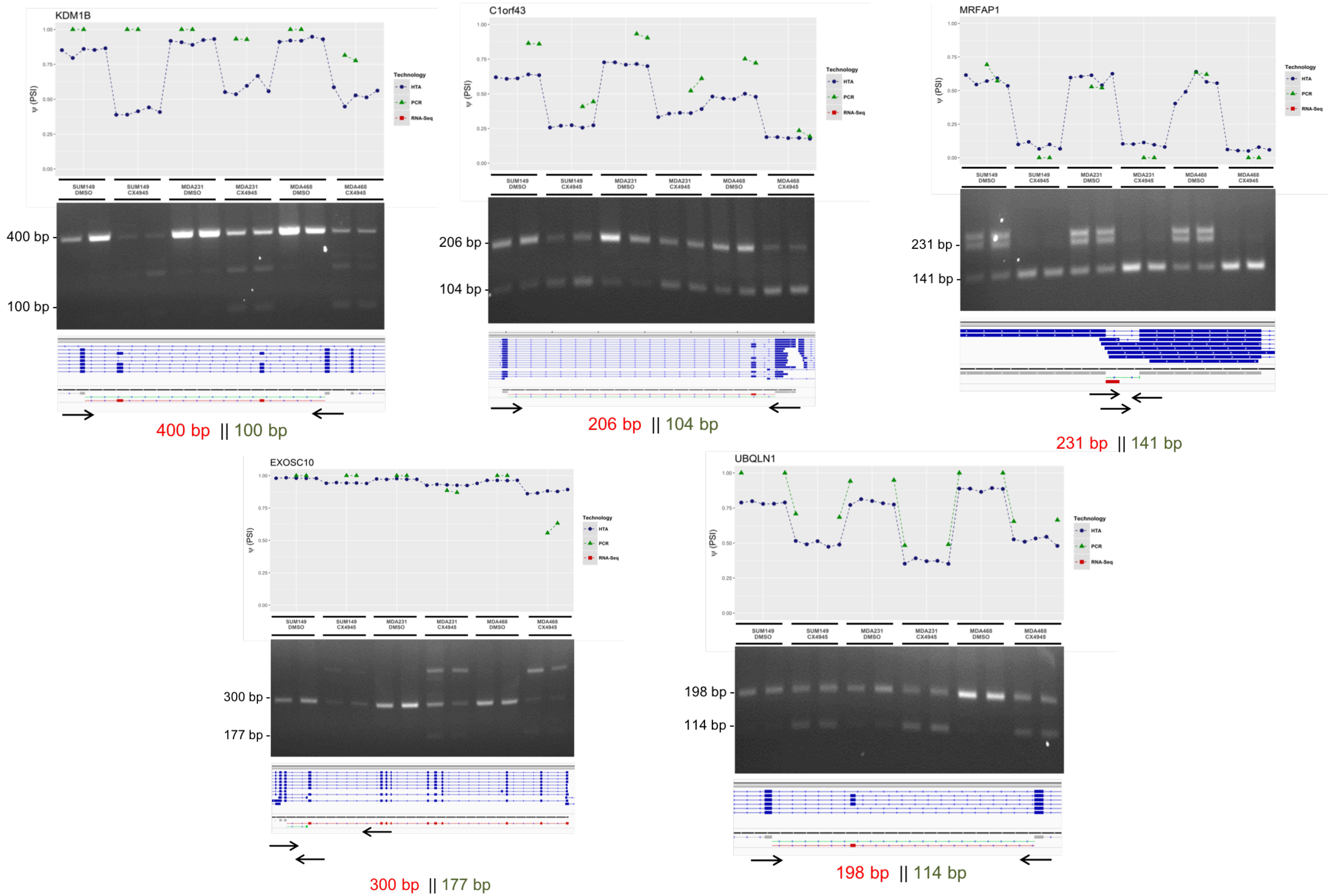

Figure S7. Estimated PSI (microarrays –blue- and PCR image analysis –green-), PCR bands, the reference HTA transcriptome and the alternative paths of the *KDM1B*, *C1orf41*, *MRFAP1*, *EXOSC10* and *UBQLN1* genes. These genes were found to be significant only by HTA. In *EXOSC10* a new unpredicted band appears. All of them were considered validated (i.e. the prediction by HTA is correct).

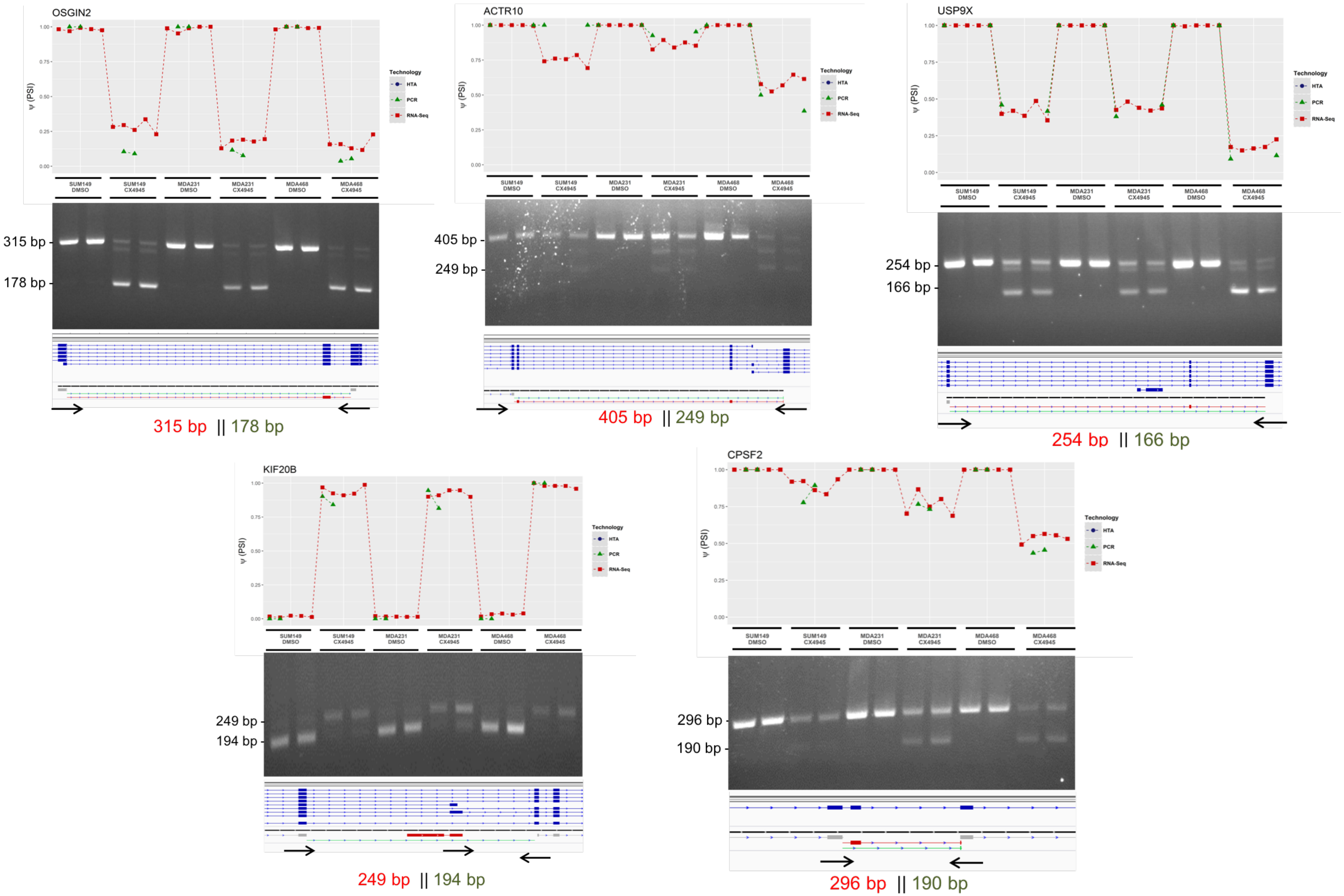

Figure S8. Estimated PSI (for RNA-seq –red- and PCR image analysis –green-), PCR bands, the reference HTA transcriptome and the alternative paths of the *OSGIN2*, *ACTR10*, *USP9X*, *KIF20B* and *CPSF2*. These genes were detected only by RNA-seq. All of them were considered validated (the PCR for *ACTR10* was noisy though).

Table S9. Design and contrast matrices provided as an input to EventPointer algorithm

Design Matrix

	Intercept	*Cell Type (MDA231)*	*Cell Type (MDA468)*	*Treatment (CX4945)*
SUM149_DMSO_1	*1*	*0*	*0*	*0*
SUM149_DMSO_2	*1*	*0*	*0*	*0*
SUM149_DMSO_3	*1*	*0*	*0*	*0*
SUM149_DMSO_4	*1*	*0*	*0*	*0*
SUM149_DMSO_5	*1*	*0*	*0*	*0*
SUM149_CX4945_1	*1*	*0*	*0*	*1*
SUM149_CX4945_2	*1*	*0*	*0*	*1*
SUM149_CX4945_3	*1*	*0*	*0*	*1*
SUM149_CX4945_4	*1*	*0*	*0*	*1*
SUM149_CX4945_5	*1*	*0*	*0*	*1*
MDA231_DMSO_1	*1*	*1*	*0*	*0*
MDA231_DMSO_2	*1*	*1*	*0*	*0*
MDA231_DMSO_3	*1*	*1*	*0*	*0*
MDA231_DMSO_4	*1*	*1*	*0*	*0*
MDA231_DMSO_5	*1*	*1*	*0*	*0*
MDA231_CX4945_1	*1*	*1*	*0*	*1*
MDA231_CX4945_2	*1*	*1*	*0*	*1*
MDA231_CX4945_3	*1*	*1*	*0*	*1*
MDA231_CX4945_4	*1*	*1*	*0*	*1*
MDA231_CX4945_5	*1*	*1*	*0*	*1*
MDA468_DMSO_1	*1*	*0*	*1*	*0*
MDA468_DMSO_2	*1*	*0*	*1*	*0*
MDA468_DMSO_3	*1*	*0*	*1*	*0*
MDA468_DMSO_4	*1*	*0*	*1*	*0*
MDA468_DMSO_5	*1*	*0*	*1*	*0*
MDA468_CX4945_1	*1*	*0*	*1*	*1*
MDA468_CX4945_2	*1*	*0*	*1*	*1*
MDA468_CX4945_3	*1*	*0*	*1*	*1*
MDA468_CX4945_4	*1*	*0*	*1*	*1*
MDA468_CX4945_5	*1*	*0*	*1*	*1*

Contrast Matrix

	DMSO vs CX4945
Intercept	*0*
Cell Type (MDA231)	*0*
Cell Type (MDA468)	*0*
Treatment (CX4945)	*1*

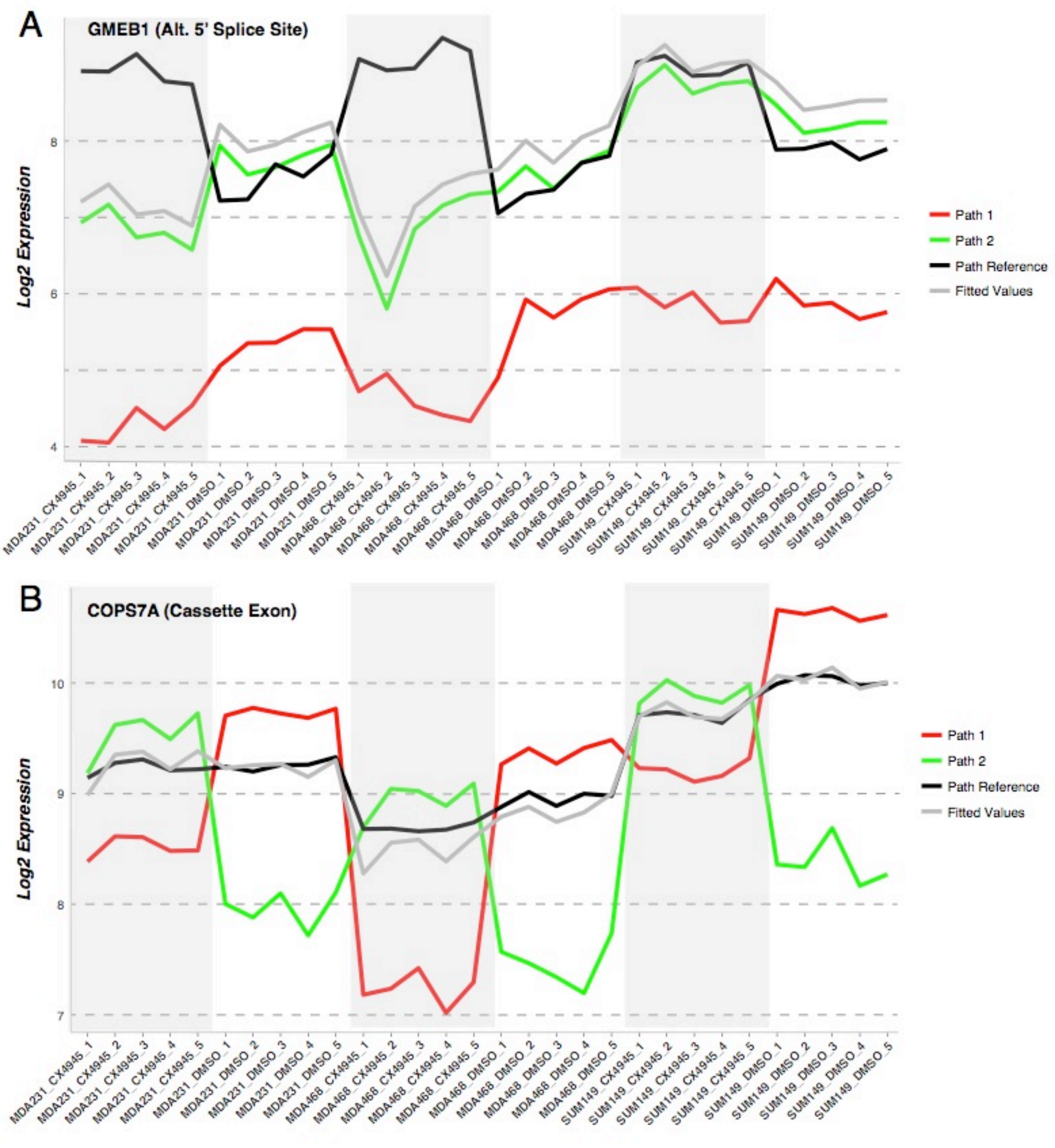

Figure S9. Summarized intensities of the paths of two alternative splicing events. A) Shows the log2 expression values of the different paths (red Path 1, green Path 2, black Path Reference) and the fitted values using non-negative least squares in gray. The fitted values are not coherent with the observed expression of the reference path. B) Log2 expression values of the different paths (red Path 1, green Path 2, black Path Reference) and the fitted values using non-negative least squares in gray. The fitted values are similar to the measured expression of the reference path.

Table S10. False discovery rate for the events using exon junction arrays. The events were separated into two groups based on the relative error. The median (0.1574) was used as the threshold to divide

	Relative Error < 0.1574	Relative Error > 0.1574
**FDR**	0.222%	0.567%
